# Morphomics, Survival, and Metabolites in Patients With Metastatic Pancreatic Cancer

**DOI:** 10.1001/jamanetworkopen.2024.40047

**Published:** 2024-10-17

**Authors:** Valerie Gunchick, Edward Brown, Juan Liu, Jason W. Locasale, Philip A. Philip, Stewart C. Wang, Grace L. Su, Vaibhav Sahai

**Affiliations:** 1 Division of Epidemiology, Department of Medicine, Vanderbilt-Ingram Cancer Center, Vanderbilt University Medical Center, Nashville, Tennessee; 2 Morphomics Analysis Group, University of Michigan, Ann Arbor; 3 Department of Surgery, University of Michigan, Ann Arbor; 4 Department of Pharmacology and Cancer Biology, Duke University School of Medicine, Durham, North Carolina; 5 Department of Oncology, Wayne State University, School of Medicine, Karmanos Cancer Center, Detroit, Michigan; 6 Division of Gastroenterology and Hepatology, University of Michigan, Ann Arbor; 7 Division of Hematology and Oncology, Department of Internal Medicine, University of Michigan, Ann Arbor; 8 University of Michigan Rogel Cancer Center, Ann Arbor; 9 Gastroenterology Section, Veterans Administration Ann Arbor Healthcare System, Ann Arbor, Michigan

## Abstract

**Importance:**

Associations of body mass index (BMI) with survival in pancreatic ductal adenocarcinoma (PDA) have substantial variability in literature, potentially due to heterogeneous patient populations and retrospective analyses. Additionally, BMI may inadequately describe body composition (ie, morphomics; including subcutaneous and visceral fats, muscle, and fascia), which might have independent biological roles and associations with survival.

**Objective:**

To study the associations of BMI and morphomics with survival and metabolomics in metastatic PDA.

**Design, Setting, and Participants:**

This cohort study prospectively collected patient data, imaging, and serum on the phase 3 trial (Avenger500), which investigated the efficacy and safety of 5-fluorouracil, leucovorin, oxaliplatin, and irinotecan (FOLFIRINOX) versus modified FOLFIRINOX plus devimistat. The randomized trial accrued 528 patients with chemotherapy-naive, metastatic PDA from Europe, Israel, Korea, and the US between 2018 and 2020. In the present study, per-protocol patients with L1 to L4, T10 to T12 vertebral levels were evaluated. Data analysis occurred from January 2023 to April 2024.

**Exposure:**

Patient data were collected by clinical staff. Morphomics were analyzed from baseline imaging. Metabolites were extracted from baseline serum.

**Main Outcome and Measures:**

A multifaceted statistical approach evaluated associations of BMI and morphomics with progression-free survival (PFS) and overall survival (OS). Associations of morphomics with metabolites were also studied.

**Results:**

Of the 528 initial patients, 476 (median [IQR] age, 63 [56-68] years; 280 male [58.8%]; median [IQR] BMI, 25.0 [22.1-25.9]) were evaluable for the present study. BMI (obese [≥30] compared with normal [18.5-24.9]) was not associated with OS (hazard ratio [HR], 0.90; 95% CI, 0.67-1.22; *P *for trend = .33). More subcutaneous fat was associated with longer OS (HR, 0.62; 95% CI, 0.41-0.94; *P* for trend = .02). Higher visceral fat density was associated with shorter PFS (HR, 1.74; 95% CI, 1.23-2.48; *P* for trend = .002) and OS (HR, 1.50; 95% CI, 1.12-2.00; *P* for trend = .008). A higher muscle-to-fascia ratio was associated with longer PFS (HR, 0.58; 95% CI, 0.40-0.84; *P* for trend = .005) and OS (HR, 0.56; 95% CI, 0.41-0.75; *P* for trend = 1.7 × 10^−4^). Subcutaneous fat was positively associated with long-chain fatty acid metabolism including pristanic acid, decanoylcarnitine, decenoylcarnitine, and octanoylcarnitine. Muscle-to-fascia was positively associated with metabolites including acetylcarnosine (β = 0.34; 95% CI, 0.21-0.47; *P* = 1.27 × 10^−6^).

**Conclusions and Relevance:**

In cohort study of patients with metastatic PDA, BMI was not associated with survival. Higher visceral fat density, subcutaneous fat area, and muscle-to-fascia ratio were associated with survival independent of BMI. The latter 2 were associated with higher levels of animal product metabolism. These findings could represent novel focuses for prognostication and intervention to improve survival of patients with PDA.

## Key Points

**Question:**

What are the associations of body mass index (BMI) and body composition (morphomics), such as fat, muscle, and fascia mass or distribution with survival for patients with metastatic pancreatic cancer?

**Findings:**

In this cohort study of 476 patients with metastatic pancreatic cancer, BMI was not associated with survival; however, multiple morphomic factors were significantly associated with progression-free and overall survival and were also associated with metabolites, including animal product metabolism and the phytanic acid pathway.

**Meaning:**

These findings suggest that, while BMI is not associated with survival among patients with pancreatic cancer, morphomics are associated with survival and metabolism; thus, these results may be used to identify potential pancreatic cancer prognostic factors and possible points for intervention to improve survival among patients with pancreatic cancer.

## Teaser

This cohort study analyzes associations of body composition variables with metabolites and survival outcomes among patients with metastatic pancreatic cancer.

## Introduction

It is estimated 66 440 people will receive a diagnosis of pancreatic cancer in 2024 and 51 750 will die.^1^ Chemotherapy improves prognosis, yet median survival remains low for patients with metastatic disease at 11.1 months.^2,3^ There are few prognosticators and even fewer interventions to improve patient survival in conjunction with chemotherapy.

Obesity is associated with an increased risk of the development of multiple cancers including pancreatic ductal adenocarcinoma (PDA). However, after cancer diagnosis, high body mass index (BMI) has been associated with longer survival in PDA.^4^ This contradiction has been coined the obesity paradox, although its validity remains uncertain. Evidence suggests the protective associations of high BMI after diagnosis could be due to statistical oversight including reverse causation and collider bias,^5^ and PDA studies have shown heterogenous associations.^4,6,7,8^ Alternatively, BMI may inadequately account for the distribution of body components, such as fat and muscle, which varies between patients. Due to limited prospective data, a clear association of BMI with PDA survival remains elusive.

Many patients with pancreatic cancer are affected by cachexia, defined as the ongoing muscle loss with or without fat loss due to their chronic illness.^9^ A small retrospective study^10^ of patients with PDA suggested fat loss is the primary force for worse survival as opposed to combined fat and muscle loss. Transcriptomic analysis indicated distinct genes cause fat and muscle loss and, therefore, fat and muscle loss likely have unique associations with PDA.^11^ While specific correlations with health are currently unknown, general evidence indicates that body compositions, or morphomics, are associated with survival in healthy adults. This includes positive associations of risk of mortality with higher visceral fat,^12,13^ whereas inverse associations have been reported with subcutaneous fat.^12,13^ Based on these data, systematic evaluation of survival among patients with PDA and morphomic variables may delineate independent roles of fat and muscle.

Few studies have evaluated morphomic variables in patients with PDA, and most are limited to retrospective, single-center evaluations with small sample sizes, while some do not include patients with advanced PDA.^14,15,16,17,18,19,20,21,22,23^ While these studies did not provide biological rationale, in other cancers and healthy populations, metabolites have been shown to be distinctly associated with body compositions, including visceral fat being associated with high levels of triglycerides and inflammation.^24,25^ Establishing biological context for associations of survival with body compositions in patients with PDA could help us identify prognosticators and opportunities for intervention to improve survival.

To address these literature gaps we identified a large, homogeneous patient population with metastatic PDA enrolled in a prospective, global, phase 3 randomized clinical trial for first-line chemotherapy. Overall, we aimed to study the association of BMI with patient survival, granularly evaluate associations of body composition variables with survival, and investigate the associations of metabolites with specific body compositions.

## Methods

### Patient Characteristics

The reporting of this cohort study followed Strengthening the Reporting of Observational Studies in Epidemiology (STROBE) reporting guideline.^26^ Patients in the Avenger500 phase 3 clinical trial,^27^ which enrolled from 2018 to 2020, were evaluated for inclusion. In total, 528 patients with metastatic PDA with no prior systemic chemotherapy for metastatic disease were randomized to 5-fluorouracil, leucovorin, oxaliplatin, and irinotecan (FOLFIRINOX) or modified FOLFIRINOX plus devimistat across 74 sites in Europe, Israel, Korea, and the US.^27^ All patients provided written informed consent to data use for research. The trial, and subsequent research, were approved by the ethics and institutional review board at each site. No difference was reported in median progression-free survival (PFS; 8.0 months; 95% CI, 7.2-11.1 months vs 7.8 months; 95% CI, 7.0-10.9 months), median (IQR) overall survival (OS; 11.7 [10.1-13.2] vs 11.1 [10.1-13.2] months), or overall response rate (34% vs 39%) between the control and experimental groups, respectively; thus, treatment group was not adjusted for in our analyses. Demographics, clinical variables, and anthropometrics were collected at baseline (after diagnosis and before therapy initiation). BMI was calculated as weight in kilograms divided by height in meters squared.^26^

### Body Composition Measurements

Baseline computed tomography (CT) scans, mandated by the study protocol, were used for morphomic analysis. Scans were completed between 45 days before to 3 days after the start of chemotherapy. Body composition measures were retrieved from CT scans using automated algorithms within the Analytic Morphomics image processing platform developed at the University of Michigan.^28,29,30^ The algorithms were programmed in MATLAB version R2022b (MathWorks) using deep learning models, as previously described, with a mean (SD) accuracy of 0.975 (0.018) compared with manually delineated morphomic areas.^31,32,33^

Seven body composition variables including subcutaneous fat, visceral fat, muscle, and fascia area and/or density were extracted as defined in eTable 1 in Supplement 1.^34^ Morphomic area variables (expressed in millimeters squared) and densities (Hounsfield units) were created by summing measurements across the T10 to T12 and L1 to L4 vertebrae for primary analysis to comprehensively represent the abdomen, as well as T10 to T12 and L3 for sensitivity analysis 1 and 4 as described below. Morphomic indexes (expressed in centimeters squared per meter squared) were calculated by dividing area (centimeters squared) by height (meters squared). Ratios were calculated by dividing morphomic area variables by fascia area to describe the morphomic variable relative to body size, as opposed to absolute measures.

### Metabolite Measurements

All 251 available US patients’ baseline serum samples were submitted for targeted metabolomic evaluation. Metabolite extractions were performed as described previously^35,36^ using liquid chromatography-mass spectrometry wherein 18 μL serum was vortexed with standards and methanol and centrifuged. Next, 5 μL supernatant supported metabolite separation implementing the hydrophilic interaction chromatography method with an Xbridge amide column (100 × 2.1 mm inner diameter [3.5 μm]). Following, metabolite profiling was completed on an Ultimate 3000 ultra-high–performance liquid chromatography (Dionex) system coupled to a Q Exactive Plus mass spectrometer (Thermo Scientific) equipped with a heated-electrospray ionization probe. Select parameters include a positive mode scan range of 70 to 900 m/z from 1.31 to 12.50 minutes and, for negative mode, a scan range of 70 to 900 m/z from 1.31 to 6.60 minutes followed by 100 to 1000 m/z from 6.61 to 12.50 minutes. Resolution was 70 000 m/z and automated gain control was targeted at 3 × 10^6^ ions. Peak extraction and integration were performed using the commercially available software Sieve version 2.2 (Thermo Scientific).

### Statistical Analysis

Characteristics of patients with PDA were evaluated with χ^2^ or Fisher exact test for categorical variables, and analysis of variance or the Kruskal-Wallis test for continuous variables, as appropriate. Cox proportional hazards models were used to estimate hazard ratios (HRs) and 95% CIs for associations of BMI and morphomic variables with PFS and OS. PFS was calculated from randomization to independent-assessor–designated disease progression or censored at the patient’s day of last radiographic tumor measurement. OS was calculated from randomization to death from any cause or censored at last follow-up. *P*-trends were evaluated using the ordinal variable. Three adjustment sets were implemented to evaluate if associations were retained despite covariate decisions: unadjusted, model 1 (adjusted for age, Eastern Cooperative Oncology Group performance status [ECOG PS], sex, diabetes, albumin, and BMI in morphomic evaluations), and model 2 (additionally adjusted for baseline carbohydrate antigen [CA]19-9 and race).

Morphomic variables were evenly divided into tertiles for associations with survival. BMI was evaluated per tertiles and World Health Organization thresholds of 18.5 to 24.9, 25.0 to 29.9, and 30.0 or greater. Patients with a BMI of 18.5 or less were excluded from BMI analyses to allow comparisons to be appropriately made with individuals with normal weight. An additional BMI analysis was evaluated within each geographic region (Israel, Korea, the US, and Europe [including Germany, Belgium, and France]). *P*-value heterogeneity tested if BMI associations varied by region.

BMI sensitivity analysis included all patients with BMI measurements (eFigure 1 in Supplement 1). Four morphomic sensitivity analyses were conducted on key morphomic variables. The first (1) contrasted primary associations with evaluations of only the T10 to T12 vertebrae. The second (2) evaluated potential collinearity effects of BMI with the morphomic variables. The third (3) ensured scan phase (venous, arterial, delayed, or noncontrast) did not affect the association of morphomic variables s with survival. The fourth (4) evaluated morphomics at only the L3 vertebrae to support the generalizability of our results to other studies. The full model was evaluated with model 1 adjustments and in sensitivity analysis 2. Proportional hazards were evaluated and, if needed, a model with an interaction between the variable and time was evaluated to ensure the association remained.

Morphomic variables with associations retained across multiple models and sensitivity analyses were selected for further analysis, as was BMI. Kaplan-Meier plots compared median survival between tertiles. Restricted cubic splines (RCS) with 5 knots and model 1 adjustments displayed association shapes. Patient characteristics were compared between the highest and lowest morphomic tertiles. Correlations between area and density were evaluated. Lastly, a full model evaluated selected morphomics variables with mutual adjustment due to the interrelatedness of body compositional measures.^37^

Metabolites with null or missing values for 30% or more of patients were excluded. Associations of metabolites with selected morphomic measurements were studied with linear regression. For linear regression models, metabolites were trimmed to 2 SDs, log-transformed (ln[x + 1]) and z-scored, and morphomic variables were z-scored. Model 1 adjustments were implemented and *P* < 6.3 × 10^−4^ was considered statistically significant after adjusting for multiple comparisons by dividing .05 by 80, the number of independent tests.^38^ All evaluations were conducted in R version 4.3.1 (R Project for Statistical Computing). Analysis was conducted from January 2023 and April 2024.

## Results

### Patient Characteristics

**Figure 1.**
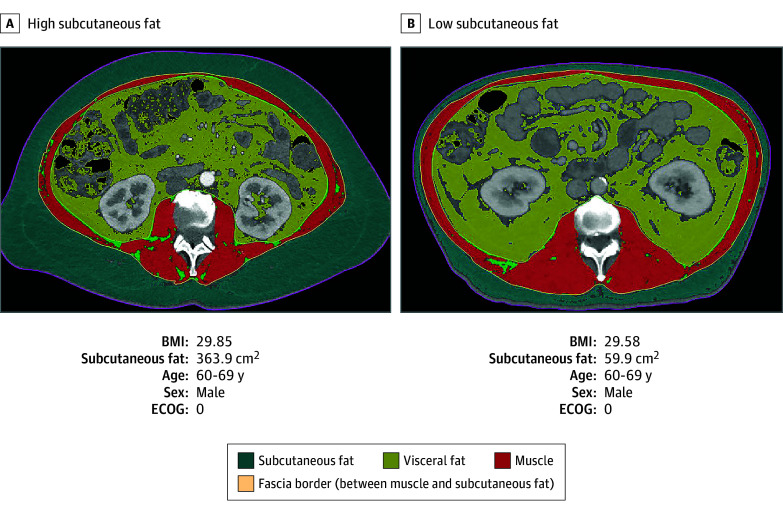
Comparison of L2 Subcutaneous Fat Between Participants With Similar Body Mass Index (BMI) BMI is calculated as weight in kilograms divided by height in meters squared. ECOG indicates Eastern Cooperative Oncology Group performance status.

Of 528 initial patients, 476 (median [IQR] age, 63 [56-68] years; 280 male [58.8%]; median [IQR] BMI, 25.0 [22.1-25.9]) were eligible for primary analysis because they underwent a baseline CT scan, had BMI recorded, received trial therapy, and their L1 to L4 and T10 to T12 vertebral measurements were included in scans (Table 1 and eFigure 1 in Supplement 1). Most were from the US (298 participants [62.6%]), and total person-time included 5351.7 months (eTable 2 in Supplement 1). A total of 219 patients had baseline serum samples for metabolomic evaluation. Patients in the high BMI tertile were more likely to be male and have more comorbidities but had similar CA19-9 and ECOG PS values compared with low and medium BMI groups (Table 1). Patients with both morphomics and metabolite data were included in metabolite evaluations (eFigure 1 in Supplement 1). Despite patients having the same sex, ECOG PS, and similar age and BMI, compositions such as subcutaneous fat varied drastically (Figure 1).

**Table 1.** Baseline Characteristics of Patients With Metastatic Pancreatic Cancer by BMI Tertile

Variable	Patients by BMI tertile, median (IQR)				*P* value
	All patients (N = 476)	Low BMI (n = 159)	Middle BMI (n = 159)	High BMI (n = 158)	
BMI^a^	25.0 (22.1-25.9)	21.5 (19.9 to 22.1)	25.0 (24.0 to 26.0)	30.4 (28.8 to 33.2)	NA
Age,y	63 (56-68)	63 (57-67)	64 (57-69)	62 (55-67)	.14
Sex, No. (%)					
Male	280 (58.8)	77 (48.4)	111 (69.8)	92 (58.2)	<.001
Female	196 (41.2)	82 (51.6)	48 (30.2)	66 (41.8)	
Baseline carbohydrate antigen19-9, U/mL	1512 (143 to 10 278)	1417 (116 to 10 000)	1961 (171 to 10 697)	1531 (136 to 10 000)	.87
ECOG PS = 1, No. (%)	244 (51.3)	90 (56.6)	75 (47.2)	79 (50.0)	.23
Baseline comorbidities, No. (%)					
Diabetes	134 (28.2)	27 (17.0)	53 (33.3)	54 (34.2)	.001
PDA resection	9 (1.9)	5 (3.1)	1 (0.6)	3 (1.9)	.26
Hypertension	225 (47.3)	56 (35.2)	73 (45.9)	96 (60.8)	<.001
Abdominal pain	278 (58.4)	87 (54.7)	93 (58.5)	98 (62.0)	.42
Hyperlipidemia	89 (18.7)	19 (11.9)	30 (18.9)	40 (25.3)	.009
Osteoarthritis	26 (5.5)	3 (1.9)	8 (5.0)	15 (9.5)	.01
Gastroesophageal reflux	137 (28.8)	36 (22.6)	38 (23.9)	63 (39.9)	.001
Albumin (normal to high)	407 (85.5)	137 (86.2)	136 (85.5)	134 (84.8)	.94
Anthropometrics					
Height, m	1.70 (1.62 to 1.78)	1.68 (1.60 to 1.75)	1.72 (1.65 to 1.79)	1.72 (1.62 to 1.78)	.001
Weight, kg	72.8 (62.1 to 85.2)	58.5 (53.8 to 64.5)	74.0 (67.0 to 80.1)	90.0 (81.0 to 102.4)	<.001
Body surface area, m^2^	1.85 (1.70 to 2.04)	1.64 (1.55 to 1.78)	1.87 (1.76 to 2.00)	2.08 (1.90 to 2.22)	<.001
Area (summed across T10-T12, L1-L4 vertebrae), cm^2^					
Muscle	7560 (6114 to 8866)	6469 (5413 to 7661)	7915 (6598 to 8876)	8582 (6865 to 9892)	<.001
Subcutaneous fat	6801 (3771 to 11 502)	3195 (18 301 to 5403)	6449 (4347 to 8616)	12 972 (10 008 to 16905)	<.001
Visceral fat	7091 (4297 to 11 909)	3535 (2164 to 5252)	7311 (5018 to 10 386)	13 037 (8880 to 16 862)	<.001
Fascia	39 112 (32 389 to 46 269)	31 759 (28 243 to 37 884)	40 717 (33 896 to 4575)	46 393 (38 688 to 53 376)	<.001
Index, cm^2^m^−2^					
Muscle	259 (226 to 290)	226 (203 to 258)	260 (235 to 285)	289 (260 to 322)	<.001
Subcutaneous fat	241 (123 to 390)	109 (63 to 208)	220 (144 to 320)	451 (325 to 615)	<.001
Visceral fat	248 (153 to 393)	125 (77 to 190)	258 (182 to 347)	432 (338 to 572)	<.001
Fascia	1333 (1175 to 1518)	1145 (1062 to 1278)	1329 (1223 to 1448)	1586 (1432 to 1766)	<.001
Density, HU					
Muscle	284 (242 to 319)	307 (277 to 339)	293 (263 to 319)	244 (207 to 283)	<.001
Subcutaneous fat	−717 (−746 to −681)	−698 (−737 to −660)	−704 (−739 to −674)	−733 (−759 to −704)	<.001
Visceral fat	−641 (−678 to −614)	−623 (−645 to −597)	−635 (−672 to −611)	−660 (−692 to −641)	<.001

Abbreviations: BMI, body mass index; ECOG PS, Eastern Cooperative Oncology Group performance status; HU, Hounsfield units; NA, not applicable; PDA, pancreatic ductal adenocarcinoma.

^a^ BMI was calculated as weight in kilograms divided by height in meters squared.

### Associations of BMI and Morphomics With Survival

#### BMI

**Figure 2.**
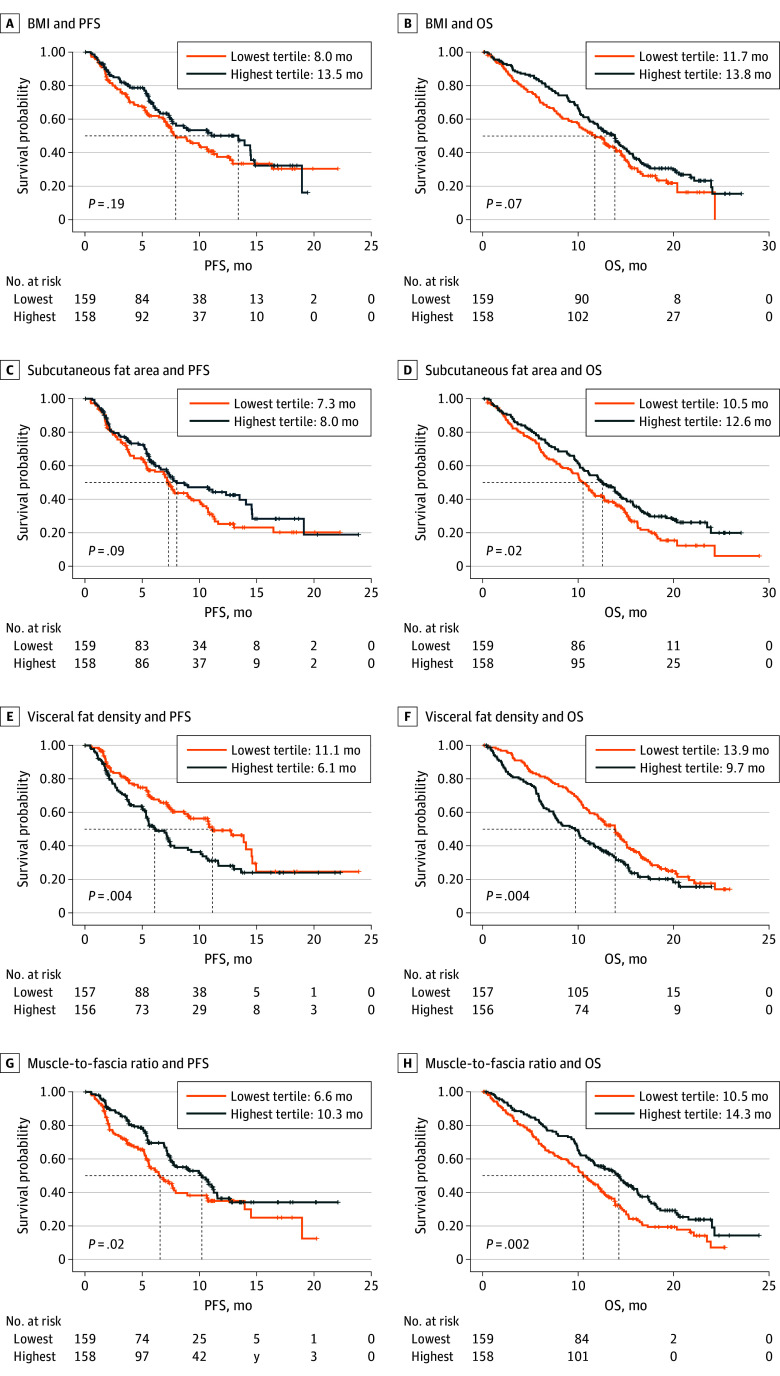
Comparison of Median Progression-Free Survival (PFS) and Overall Survival (OS) per Body Mass Index (BMI) and Selected Morphomic Variables A comparison of the highest to lowest tertile for BMI (A,B) and morphomic variables of subcutaneous fat area (C,D), visceral fat density (E,F) and muscle-to-fascia (G,H) is shown for PFS and OS. The dotted lines reflect the median.

Of the 476 patients in the analytic cohort, 18 (3.8%) had a BMI less than 18.5 and were excluded from BMI analyses. Patients with a high BMI (≥30) did not experience statistically significant longer survival than patients with a low BMI (18.5-24.9; HR for PFS, 0.97; 95% CI, 0.67-1.42; *P* for trend = .84; HR for OS, 0.90; 95 CI, 0.67-1.22; *P* for trend =.38) (eTable 3 in Supplement 1). No trends or statistically significant associations were observed between BMI and survival in tertile analysis (eTable 3 in Supplement 1), Kaplan-Meier plots (Figure 2), RCS (eFigure 2 in Supplement 1), or sensitivity analyses (eTable 4 in Supplement 1). Results from all model adjustment sets are in eTable 3 in Supplement 1.

When BMI was evaluated within each geographic region, *P*-value heterogeneities were greater than .05, supporting a lack of association of BMI with PFS and OS in the overall population and within geographically distinct patient populations (eTable 2 in Supplement 1). However, PFS and OS associations were significantly inverse for patients in Korea (eTable 2 in Supplement 1).

#### Subcutaneous Fat

Subcutaneous fat area was significantly associated with a reduced risk of death (HR for OS, 0.62; 95% CI, 0.41-0.94; *P *for trend = .02) (Table 2). The association was retained across models and sensitivity analyses (eTable 3 and eTable 4 in Supplement 1), and a similar trend was observed for PFS; subcutaneous fat area was selected for further analysis. RCS further clarified patients with low subcutaneous fat were at higher risk of death (eFigure 2 in Supplement 1). Patients in the highest subcutaneous fat area tertile were more likely to be female and have diabetes, hypertension, osteoarthritis, and gastroesophageal reflux (eTable 5 in Supplement 1). More subcutaneous fat area was associated with less dense subcutaneous fat (eFigure 3 in Supplement 1).

**Table 2.** Associations of Select Morphomic Variables With Survival

Morphomic variable	Progression-free survival, HR (95% CI)^a^				Overall survival, HR (95% CI)^a^			
	Model 1	*P *for trend	Full model	*P *for trend	Model 1	*P *for trend	Full model	*P *for trend
Subcutaneous fat area, tertile								
Low	1 [Reference]	.10	1 [Reference]	.10	1 [Reference]	.02	1 [Reference]	.007
Middle	0.58 (0.40-0.84)		0.60 (0.41-0.88)		0.65 (0.48-0.88)		0.65 (0.48-0.88)	
High	0.70 (0.42-1.18)		0.73 (0.43-1.23)		0.62 (0.41-0.94)		0.60 (0.39-0.92)	
Visceral fat density, tertile^b^								
Low	1 [Reference]	.002	1 [Reference]	6.1 × 10^−4^	1 [Reference]	.008	1 [Reference]	.004
Middle	1.15 (0.81-1.63)		1.25 (0.88-1.78)		0.94 (0.71-1.24)		1.00 (0.76-1.33)	
High	1.74 (1.23-2.48)		1.81 (1.27-2.60)		1.50 (1.12-2.00)		1.52 (1.14-2.03)	
Muscle-to-fascia ratio, tertile^b^								
Low	1 [Reference]	.005	1 [Reference]	3.7 × 10^−4^	1 [Reference]	1.7 × 10^−4^	1 [Reference]	7.2 × 10^−5^
Middle	0.66 (0.47-0.93)		0.61 (0.43-0.87)		0.68 (0.52-0.90)		0.66 (0.50-0.87)	
High	0.58 (0.40-0.84)		0.48 (0.32-0.71)		0.56 (0.41-0.75)		0.49 (0.36-0.67)	

Abbreviations: HR, hazard ratio.

^a^ HRs and 95% CIs from Cox proportional hazards models with model 1 adjustments (age, Eastern Cooperative Oncology Group performance status, sex, diabetes, albumin, and body mass index). The full model additionally adjusted for all 3 morphomic variables (total patients, 476; progression, 222 patients; death, 332 patients).

^b^ Indicates novel finding in pancreatic ductal adenocarcinoma.

#### Visceral Fat

Higher visceral fat density was associated with statistically significant higher risks of progression (HR, 1.74; 95% CI, 1.23-2.48; *P *for trend = .002) and death (HR, 1.50; 95% CI, 1.12-2.00; *P *for trend = .008 (Table 2). These associations were robust (eTable 3 and eTable 4 in Supplement 1); visceral fat density was selected for further evaluation. RCS described patients with more dense visceral fat as being at higher risk of progression and death (eFigure 2 in Supplement 1). More visceral fat area was associated with less dense visceral fat (eFigure 3 in Supplement 1).

#### Muscle and Fascia

No associations of muscle morphomics with PFS or OS were observed and retained throughout models and sensitivity analyses (eTable 3 and eTable 4 in Supplement 1). Fascia index was associated with PFS and OS, although HRs varied substantially based on covariate adjustments and in sensitivity analyses (eTable 3 and eTable 4 in Supplement 1). Neither fascia nor muscle variables were selected for further analysis.

#### Selected Ratios

Twelve ratios were evaluated, while only a higher muscle-to-fascia ratio was associated with a substantially lower risk of progression (HR, 0.58; 95% CI, 0.40-0.84; *P *for trend = .005) and death (HR, 0.56; 95% CI, 0.41-0.75; *P *for trend = 1.7 × 10^−4^) (Table 2). These associations were retained in multiple settings (eTable 3 and eTable 4 in Supplement 1).

RCS indicated patients with a low muscle-to-fascia ratio were at higher risk of progression and death (eFigure 2 in Supplement 1). Patients with a higher muscle-to-fascia ratio were more likely to be younger; male; and without diabetes, hypertension, osteoarthritis, or gastroesophageal reflux (eTable 5 in Supplement 1).

#### Full Model

Associations of the key morphomics with PFS and OS had increased magnitude after mutual adjustment (Table 1). Significant results were retained in sensitivity analysis.

### Associations of Metabolites and Morphomics

**Figure 3.**
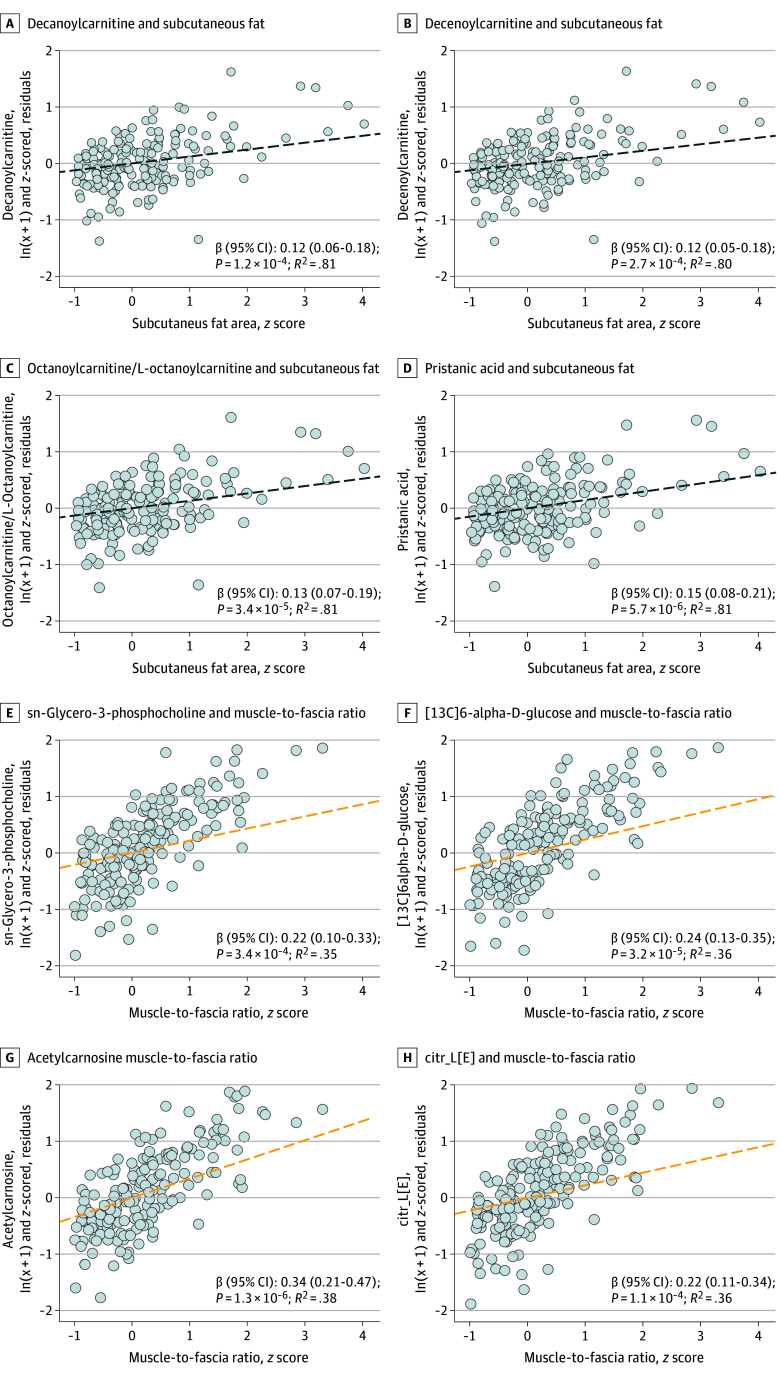
Significant Associations of Key Morphomic Variables With Metabolites Multivariate linear regression model results of metabolites and morphomic variables with models adjusted for age, Eastern Cooperative Oncology Group performance status, sex, body mass index, albumin, and diabetes.

Of 200 metabolites, 4 (pristanic acid, decanoylcarnitine, decenoylcarnitine, and octanoylcarnitine) were associated (significance threshold of *P* < 6.3 × 10^−4^) with subcutaneous fat area (Figure 3). The largest-magnitude association was for pristanic acid (β = 0.15; 95% CI,0.08-0.21; *P* = 5.7 × 10^−6^), and 81% of subcutaneous fat variance was described by our metabolite model. Four metabolites were positively associated with the muscle-to-fascia area ratio and the largest-magnitude association was for acetylcarnosine (β = 0.34; 95% CI, 0.21-0.47; *P* = 1.27 × 10^−6^), and 38% of muscle-to-fascia ratio variance was described by our metabolite model. No metabolites were associated with visceral fat density at *P* < 6.3 × 10^−4^ (eFigure 4 in Supplement 1).

## Discussion

In this large cohort study, we observed no association of BMI with survival for patients with metastatic PDA. However, longer survival was associated with more subcutaneous fat and a higher muscle-to-fascia ratio, whereas shorter survival was associated with more dense visceral fat. In addition, we observed large-magnitude associations of subcutaneous fat and muscle-to-fascia ratio with several metabolites, which provide key biological insight.

Published studies of BMI and survival among patients with PDA report conflicting results ranging from inverse^4^ to positive.^6,7^ The positive associations support the obesity paradox wherein reverse causality indicates patients with higher BMIs live longer than sicker patients with very low BMIs.^5^ This notion is substantiated by a retrospective study^4^ observing a positive association in which 43% of patients with underweight received chemotherapy compared with 60.5% of patients with overweight (*P* < .001). The null association in our prospective study supports a 10-study meta-analysis comparing patients with PDA with overweight with healthy patients.^8^

Prior evaluations of subcutaneous fat and death in retrospective, single-site populations have reported null,^14,15,16^ inverse,^23^ and positive^20^ associations, whereas we observed an inverse association. High subcutaneous fat may represent a lack of cachexia,^39^ which is associated with worse PDA nutritional status, lung function, and survival.^40^ Alternatively, subcutaneous fat could serve as an energy reservoir during decreased caloric intake, which is common in PDA.

High subcutaneous fat area was associated with more pristanic acid, an intermediate in α-oxidation of phytanic acid.^41,42,43^ High subcutaneous fat was also associated with higher concentrations of decanoylcarnitine, decenoylcarnitine, and octanoylcarnitine; all are involved in long-chain fatty acid metabolism, including in the phytanic acid pathway. Phytanic acid primarily enters the body in animal food products,^44^ particularly from cows,^45,46^ as opposed to plants because the human gut inefficiently absorbs its precursor chlorophyll.^47,48^ While red meat is considered a carcinogen^49^ and generally deleterious for health, our data indicate higher baseline animal product intake was associated with higher subcutaneous fat area and may be associated with longer survival.

To our knowledge, we are the first to report associations of more dense visceral fat with shorter PFS and OS in PDA. Similar associations of visceral fat density have been observed in hepatocellular,^50^ esophageal,^51^ and colorectal cancers.^52^ Visceral fat density may be associated with shorter survival due to proximity because direct contact between adipocytes and cancer cells has been shown to induce robust tumoral invasion.^53^ Further, dense fat is comprised of increased, smaller adipocytes,^54^ and a higher ratio of adipocytes to tumor cells has been positively associated with invasion.^53^ More dense fat may result from poor fat quality, including increased fibrosis and inflammation,^55^ or lower lipid content from shrunken adipocytes from cachexia. Visceral fat density may allude to disease prognosis independent of known prognosticators such as disease stage and ECOG PS.

The association of a higher muscle-to-fascia ratio with longer PFS and OS is another novel finding. Muscle has been shown to causally improve survival in a clinical trial of 65 patients with pancreaticobiliary cancer randomized to participate in resistance training plans.^56^ The metabolite with the largest-magnitude association with the muscle-to-fascia ratio was acetylcarnosine, an acylated version of carnosine (β-alanine-histidine) resistant to carnosinase, which maintains carnosine for use in the body.^57^ Carnosine is found in muscle and is primarily consumed from diet,^58,59^ mostly easily from cows because their muscle contains 3 times the amount of carnosine as poultry.^60^ Carnosine can be synthesized, but is limited by β-alanine availability.^58,59^ Carnosine has anticancer properties^60,61^ and has been shown to inhibit prostate,^62^ colorectal,^63^ breast, ovarian, colon, and leukemia cancer cell proliferation.^61^ Carnosine was not directly measured in our dataset precluding direct analysis. Together, these data suggest that patients with more muscle relative to abdominal compartment size (fascia) at baseline lived longer and had higher baseline levels of acetylcarnosine.

Our study has multiple advantages over previous studies. First, we utilized the largest morphomics dataset in PDA and our cohort only included patients with treatment-naive metastatic PDA. Second, our data quality was robust due to prospective collection and monitoring required by the Food and Drug Administration for a phase 3 clinical trial.^19,20,21,22^ Third, to our knowledge, we are the first to evaluate morphomic variable associations with PFS. Fourth, we removed patients with underweight in BMI evaluations, thereby mitigating effects of the obesity paradox. Fifth, we studied baseline metabolites, thereby avoiding biases present in other studies due to studying prediagnosis metabolites.^64,65,66^

### Limitations

Potential limitations of our study include selection bias because obesity is associated with increased risk of pancreatic cancer and death.^67^ Additionally, we were unable to adjust for cachexia^9^ or sarcopenia,^68^ which may introduce bias into our results. Our results are likely generalizable to patients with PDA with an ECOG PS of 0 to 1, who are treatment-naive for metastatic PDA, and with an expected survival of more than 3 months; additional studies are needed in other PDA populations.

## Conclusions

In summary, in this cohort study BMI was not associated with survival among patients with metastatic PDA. Independent of BMI, we observed more subcutaneous fat area and a higher muscle-to-fascia ratio to be associated with longer survival, both of which were associated with higher levels of animal meat and dairy metabolism. More dense visceral fat was associated with shorter survival. Our findings represent novel focuses for patient survival prognostication and potential interventions to improve survival among patients with PDA.

## Notes

Supplement 1. **eTable 1**. Morphomic Variable Specifics **eFigure 1.** CONSORT Diagram of Analysis Groups **eTable 2.** Associations of Body Morphomic Index and Survival by Region **eTable 3.** Associations Between BMI and Morphomic Variables With Progression-Free and Overall Survival **eFigure 2.** Association Shapes of BMI and Morphomic Variables With Progression-Free and Overall Survival **eTable 4.** Sensitivity Analyses **eTable 5.** Comparing Patient Characteristics Between the Highest and Lowest Morphomic Variable Tertiles **eFigure 3.** Association of Key Morphomic Variables’ Area and Density Variables **eFigure 4.** Associations of Metabolites and Morphomic Variables

Supplement 2.
Data Sharing Statement
