# Supplementary material for: Morphomics, Survival, and Metabolites in Patients With Metastatic Pancreatic Cancer
Source: JAMA Netw Open. 2024 Oct 17;7(10):e2440047. doi: 10.1001/jamanetworkopen.2024.40047 (PMC11581562; doi:10.1001/jamanetworkopen.2024.40047)
Supplement: Supplement 1. — eTable 1. Morphomic Variable Specifics eFigure 1. CONSORT Diagram of Analysis Groups eTable 2. Associations of Body Morphomic Index and Survival by Region eTable 3. Associations Between BMI and Morphomic Variables With Progression-Free and Overall Survival eFigure 2. Association Shapes of BMI and Morphomic Variables With Progression-Free and Overall Survival eTable 4. Sensitivity Analyses eTable 5. Comparing Patient Characteristics Between the Highest and Lowest Morphomic Variable Tertiles eFigure 3. Association of Key Morphomic Variables’ Area and Density Variables eFigure 4. Associations of Metabolites and Morphomic Variables [file jamanetwopen-e2440047-s001.pdf]

Supplemental Online Content

Gunchick V, Brown E, Liu J, et al. Morphomics, survival, and metabolites in patients with metastatic pancreatic cancer. *JAMA Netw. Open*. 2024;7(10):e2440047. doi:10.1001/jamanetworkopen.2024.40047

eTable 1. Morphomic Variable Specifics

eFigure 1. CONSORT Diagram of Analysis Groups

eTable 2. Associations of Body Morphomic Index and Survival by Region

eTable 3. Associations Between BMI and Morphomic Variables With Progression-Free and Overall Survival

eFigure 2. Association Shapes of BMI and Morphomic Variables With Progression-Free and Overall Survival

eTable 4. Sensitivity Analyses

eTable 5. Comparing Patient Characteristics Between the Highest and Lowest Morphomic Variable Tertiles

eFigure 3. Association of Key Morphomic Variables' Area and Density Variables

eFigure 4. Associations of Metabolites and Morphomic Variables

This supplemental material has been provided by the authors to give readers additional information about their work.

© 2024 Gunchick V et al. *JAMA Network Open.*

eTable 1. Morphomic variable specifics

Variable	Definition	Lowest Tertile	Middle Tertile	Highest Tertile
		N or Median [25%, 75%]		
**BMI** (kgm^{-2}) *<18.5 excluded*		153<br>21.8 [20.6, 22.6]	153<br>25.1 [24.2, 26.3]	152<br>30.5 [28.9, 33.2]
**Muscle**				
Area (cm^2)	Area of skeletal muscle (psoas, erector spinae, quadratus lumborum, transverse abdominus, rectus abdominus, internal and external obliques)	159<br>5659 [5307, 6112]	159<br>7574 [7107, 7994]	158<br>94756 [8870, 10195]
Index (cm^{2}m^{-2})	Area divided by height (m^2)	159<br>213.2 [198.1, 226.1]	159<br>258.9 [249.9, 268.8]	158<br>305.2 [290.6, 327.5]
Density (HU)	Density: median pixel intensity of area; patient omitted if had <200 fat pixels	159<br>223 [193, 242]	159<br>285 [274, 297]	158<br>338 [319, 353]
**Subcutaneous fat**				
Area (cm^2)	Area between skin and fascia (abdominal compartment) meeting fat density thresholds (-205 to -51 HU)	159<br>2702 [1748, 3763]	159<br>6812 [5977, 8001]	158<br>13248 [11518, 16996]
Index (cm^{2}m^{-2})	Area divided by height (m^2)	159<br>93.4 [57.6, 122.9]	159<br>242.4 [206.0, 272.4]	158<br>463.2 [391.4, 625.6]
Density (HU)	Density: median pixel intensity of area; patient omitted if had <200 fat pixels	141<br>-762 [-782, -746]	141<br>-717 [-728, -703]	141<br>-663 [-681, -632]
**Visceral fat**				
Area (cm^2)	Area inside fascia (abdominal compartment) meeting fat density thresholds (-205 to -51 HU)	159<br>3187 [2126, 4297]	159<br>7120 [5806, 8428]	158<br>13860 [11957, 16966]
Index (cm^{2}m^{-2})	Area divided by height (m^2)	159<br>115.7 [74.2, 152.3]	159<br>248.7 [209.3, 288.3]	158<br>447.5 [393.7, 572.0]
Density (HU)	Density: median pixel intensity of area; patient omitted if had <200 fat pixels	157<br>-691 [-713, -678]	157<br>-641 [-652, -633]	156<br>-600 [-614, -582]
**Fascia**				
Area (cm^2)	Total area inside the abdominal compartment	159<br>30389 [27582, 32387]	159<br>39117 [37409, 41440]	158<br>49328 [46284, 53388]
Index (cm^{2}m^{-2})	Area divided by height (m^2)	159<br>1129 [1047, 1175]	159<br>1333 [1296, 1394]	158<br>1635 [1520, 1771]
**Selected ratios**				
Muscle:fascia	Muscle area divided by fascia (area inside the abdominal compartment)	159<br>0.1631 [0.1514, 0.1721]	159<br>0.191 [0.1853, 0.1971]	158<br>0.2226 [0.2113, 0.2336]
Subcutaneous fat:fascia	Subcutaneous fat area divided by fascia (area inside the abdominal compartment)	159<br>0.07327 [0.04538, 0.0970]	159<br>0.1811 [0.1416, 0.2047]	158<br>0.3398 [0.2847, 0.4256]

N of primary analysis. *BMI: body mass index, HU: Hounsfield units.*

© 2024 Gunchick V et al. JAMA Network Open.

eFigure 1. Consort diagram of analysis groups

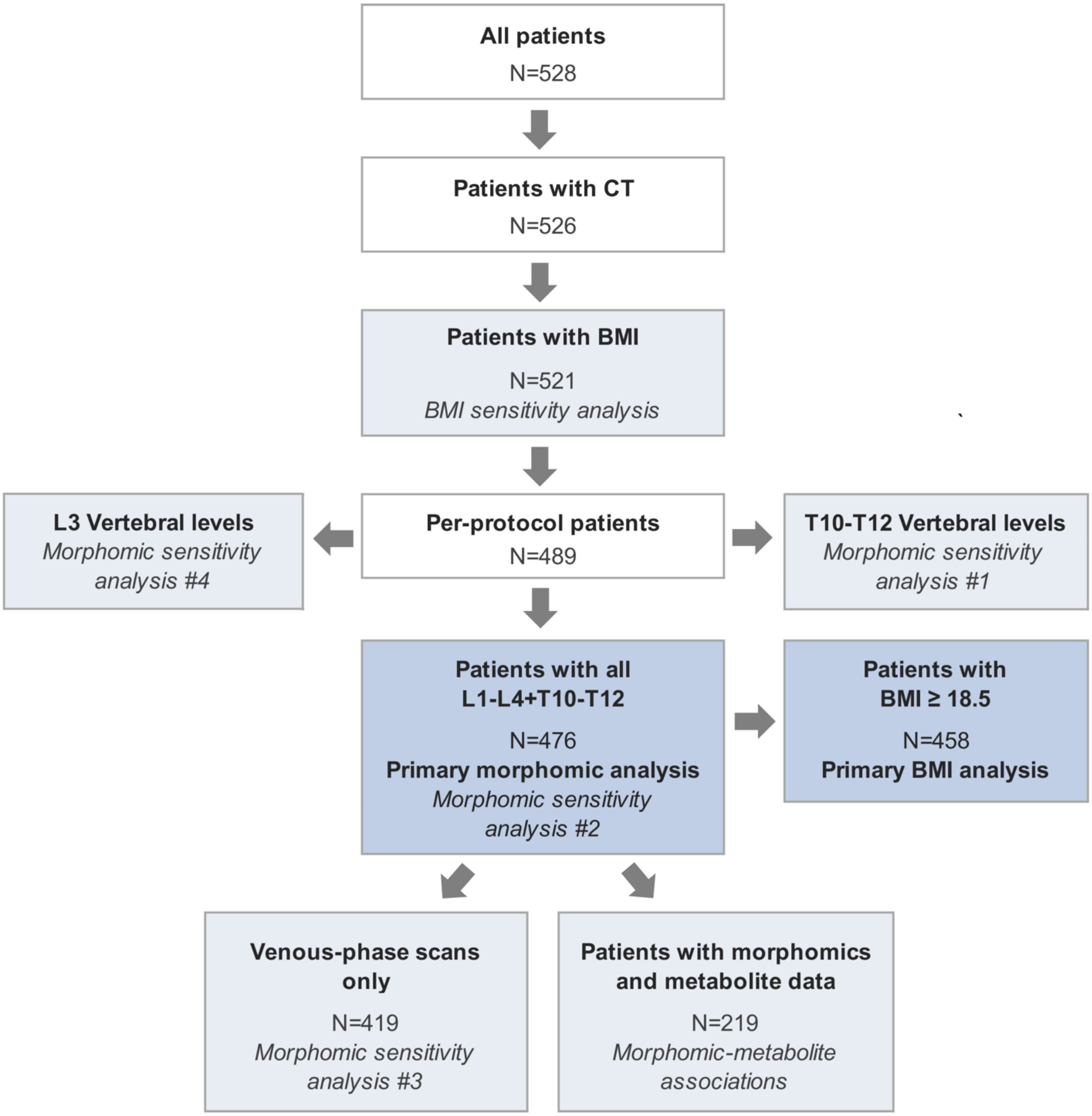

© 2024 Gunchick V et al. JAMA Network Open.

eTable 2. Associations of body morphomic index and survival by region

Region, evaluation	Low tertile	Middle tertile	High tertile	P-trend
**Europe**				
PFS HR (95% CI), p-trend:	1.00 (Reference)	0.62 (0.25, 1.52)	1.10 (0.49, 2.44)	0.83
OS HR (95% CI), p-trend:	1.00 (Reference)	1.35 (0.64, 2.86)	1.59 (0.77, 2.62)	0.21
N	29	28	28	
BMI Median [25%, 75%]	21.2 [19.9, 21.6]	23.4 [23.0, 24.0]	27.6 [25.5, 31.1]	
**Israel**				
PFS HR (95% CI), p-trend:	1.00 (Reference)	1.60 (0.49, 5.18)	1.01 (0.29, 3.53)	0.90
OS HR (95% CI), p-trend:	1.00 (Reference)	0.71 (0.20, 2.61)	1.32 (0.42, 4.17)	0.63
N	9	8	8	
BMI Median [25%, 75%]	21.9 [19.8, 22.0]	24.5 [23.6, 24.7]	26.6 [25.6 [29.2]	
**Korea**				
PFS HR (95% CI), p-trend:	1.00 (Reference)	0.42 (0.14, 1.24)	0.29 (0.09, 0.96)	0.051
OS HR (95% CI), p-trend:	1.00 (Reference)	0.38 (0.15, 0.98)	0.32 (0.12, 0.82)	0.03
N	17	17	16	
BMI Median [25%, 75%]	20.9 [20.5, 22.1]	23.2 [22.9, 23.6]	25.9 [25.2, 27.2]	
**USA**				
PFS HR (95% CI), p-trend:	1.00 (Reference)	1.06 (0.69, 1.62)	0.98 (0.64, 1.51)	0.93
OS HR (95% CI), p-trend:	1.00 (Reference)	0.83 (0.60, 1.17)	0.84 (0.60, 1.18)	0.32
N	100	99	99	
BMI Median [25%, 75%]	22.2 [21.2, 23.3]	26.7 [25.5, 27.8]	32.0 [30.2, 34.5]	
**PFS p-heterogeneity**	0.31			
**OS p-heterogeneity**	0.07			

Hazard ratios (HRs) and 95% confidence intervals (CI) from multivariate Cox proportional models. Model adjustments include age, ECOG PS, sex, diabetes, and albumin. Europe includes Germany, Belgium, and France. *BMI: Body mass index, ECOG PS: Eastern Cooperative Oncology Group performance status.*

© 2024 Gunchick V et al. JAMA Network Open.

eTable 3. Associations between BMI and morphomic variables with progression-free and overall survival

			Progression-free survival<br>HR (95% CI), p-trend				Overall survival<br>HR (95% CI), p-trend			
			**18.5-24.9, 25.0-24.9, ≥30**							
BMI		*Unadjusted:*	1.00 (Reference)	0.94 (0.69, 1.28)	0.90 (0.62, 1.30)	0.54	1.00 (Reference)	0.86 (0.67, 1.11)	0.85 (0.63, 1.15)	0.22
		Model 1:	1.00 (Reference)	0.96 (0.71, 1.31)	0.97 (0.67, 1.42)	0.84	1.00 (Reference)	0.88 (0.68, 1.13)	0.90 (0.67, 1.22)	0.38
		Model 2:	1.00 (Reference)	0.88 (0.64, 1.21)	0.97 (0.66, 1.44)	0.71	1.00 (Reference)	0.83 (0.63, 1.08)	0.90 (0.65, 1.24)	0.33
			**Low, middle, high tertiles**							
		Unadjusted:	1.00 (Reference)	1.10 (0.80, 1.52)	0.85 (0.61, 1.19)	0.36	1.00 (Reference)	0.97 (0.74, 1.28)	0.85 (0.65, 1.12)	0.24
		Model 1:	1.00 (Reference)	1.06 (0.77, 1.48)	0.86 (0.61, 1.21)	0.39	1.00 (Reference)	0.98 (0.74, 1.29)	0.87 (0.67, 1.15)	0.33
		Model 2:	1.00 (Reference)	0.99 (0.71, 1.40)	0.86 (0.61, 1.23)	0.42	1.00 (Reference)	0.91 (0.68, 1.22)	0.87 (0.66, 1.15)	0.33
**Muscle**										
	Area	*Unadjusted:*	1.00 (Reference)	0.93 (0.67-1.28)	0.97 (0.70-1.35)	0.88	1.00 (Reference)	1.04 (0.80-1.36)	1.04 (0.80-1.35)	0.80
		Model 1:	1.00 (Reference)	0.69 (0.43-1.11)	0.63 (0.34-1.16)	0.19	1.00 (Reference)	0.92 (0.63-1.34)	0.88 (0.53-1.44)	0.62
		Model 2:	1.00 (Reference)	0.75 (0.45-1.24)	0.73 (0.37-1.42)	0.45	1.00 (Reference)	0.97 (0.65-1.45)	1.04 (0.61-1.76)	0.83
	Index	*Unadjusted:*	1.00 (Reference)	0.81 (0.59-1.12)	0.86 (0.62-1.19)	0.35	1.00 (Reference)	0.79 (0.61-1.04)	0.88 (0.68-1.14)	0.35
		Model 1:	1.00 (Reference)	0.78 (0.54-1.12)	0.69 (0.44-1.07)	0.09	1.00 (Reference)	0.75 (0.56-1.02)	0.71 (0.49-1.03)	0.07
		Model 2:	1.00 (Reference)	0.87 (0.59-1.28)	0.79 (0.50-1.26)	0.33	1.00 (Reference)	0.83 (0.61-1.14)	0.82 (0.56-1.20)	0.30
	Density	*Unadjusted:*	1.00 (Reference)	0.98 (0.71-1.34)	0.76 (0.55-1.05)	0.10	1.00 (Reference)	1.06 (0.82-1.38)	0.85 (0.66-1.11)	0.24
		Model 1:	1.00 (Reference)	0.79 (0.56-1.13)	0.63 (0.42-0.94)	0.02	1.00 (Reference)	0.93 (0.70-1.24)	0.76 (0.55-1.06)	0.10
		Model 2:	1.00 (Reference)	0.85 (0.59-1.21)	0.67 (0.44-1.03)	0.06	1.00 (Reference)	0.97 (0.73-1.31)	0.80 (0.56-1.13)	0.21
**Subcutaneous fat**										
	Area	*Unadjusted:*	1.00 (Reference)	0.58 (0.42-0.81)	0.77 (0.56-1.04)	0.07	1.00 (Reference)	0.69 (0.53-0.90)	0.73 (0.56-0.94)	0.02
		Model 1:	1.00 (Reference)	0.58 (0.40-0.84)	0.70 (0.42-1.18)	0.10	1.00 (Reference)	0.65 (0.48-0.88)	0.62 (0.41-0.94)	0.02
		Model 2:	1.00 (Reference)	0.56 (0.38-0.82)	0.68 (0.40-1.16)	0.08	1.00 (Reference)	0.62 (0.45-0.86)	0.59 (0.38-0.91)	0.01
	Index	*Unadjusted:*	1.00 (Reference)	0.62 (0.44-0.86)	0.83 (0.61-1.14)	0.22	1.00 (Reference)	0.75 (0.57-0.97)	0.77 (0.59-0.99)	0.04
		Model 1:	1.00 (Reference)	0.65 (0.44-0.94)	0.82 (0.48-1.39)	0.30	1.00 (Reference)	0.72 (0.53-0.97)	0.66 (0.43-1.01)	0.045
		Model 2:	1.00 (Reference)	0.61 (0.41-0.90)	0.84 (0.49-1.44)	0.35	1.00 (Reference)	0.68 (0.50-0.94)	0.66 (0.43-1.02)	0.049
	Density	*Unadjusted:*	1.00 (Reference)	1.20 (0.84-1.71)	1.21 (0.85-1.73)	0.30	1.00 (Reference)	0.95 (0.72-1.26)	0.95 (0.72-1.26)	0.72
		Model 1:	1.00 (Reference)	1.17 (0.81-1.69)	1.34 (0.91-1.96)	0.14	1.00 (Reference)	0.90 (0.68-1.20)	0.93 (0.69-1.26)	0.63
		Model 2:	1.00 (Reference)	1.26 (0.87-1.83)	1.44 (0.96-2.15)	0.07	1.00 (Reference)	0.90 (0.67-1.22)	0.91 (0.66-1.25)	0.53
**Visceral fat**										
	Area	*Unadjusted:*	1.00 (Reference)	0.69 (0.50-0.96)	0.97 (0.71-1.33)	0.80	1.00 (Reference)	0.82 (0.63-1.07)	1.00 (0.77-1.29)	0.99
		Model 1:	1.00 (Reference)	0.67 (0.47-0.95)	0.99 (0.62-1.56)	0.71	1.00 (Reference)	0.84 (0.63-1.13)	1.06 (0.72-1.55)	0.88
		Model 2:	1.00 (Reference)	0.64 (0.44-0.92)	0.95 (0.59-1.54)	0.62	1.00 (Reference)	0.78 (0.57-1.06)	1.03 (0.69-1.53)	0.97
	Index	*Unadjusted:*	1.00 (Reference)	0.77 (0.56-1.07)	1.02 (0.74-1.39)	0.96	1.00 (Reference)	0.90 (0.69-1.17)	0.95 (0.73-1.24)	0.72
		Model 1:	1.00 (Reference)	0.80 (0.56-1.14)	1.12 (0.71-1.77)	0.73	1.00 (Reference)	0.93 (0.69-1.24)	1.00 (0.69-1.46)	0.97
		Model 2:	1.00 (Reference)	0.75 (0.51-1.10)	1.12 (0.69-1.80)	0.72	1.00 (Reference)	0.84 (0.61-1.14)	0.95 (0.63-1.41)	0.75
	Density	*Unadjusted:*	1.00 (Reference)	1.10 (0.79-1.55)	1.61 (1.16-2.24)	0.004	1.00 (Reference)	0.96 (0.73-1.26)	1.46 (1.13-1.90)	0.006
		Model 1:	1.00 (Reference)	1.15 (0.81-1.63)	1.74 (1.23-2.48)	0.002	1.00 (Reference)	0.94 (0.71-1.24)	1.50 (1.12-2.00)	0.008
		Model 2:	1.00 (Reference)	1.12 (0.78-1.62)	1.86 (1.29-2.70)	$9.0x10^{-4}$	1.00 (Reference)	0.90 (0.67-1.20)	1.53 (1.14-2.07)	0.007
**Fascia**										
	Area	*Unadjusted:*	1.00 (Reference)	0.94 (0.68-1.30)	1.20 (0.87-1.65)	0.28	1.00 (Reference)	0.95 (0.73-1.24)	1.13 (0.87-1.46)	0.37
		Model 1:	1.00 (Reference)	1.06 (0.70-1.62)	1.56 (0.89-2.73)	0.09	1.00 (Reference)	1.03 (0.73-1.45)	1.35 (0.86-2.11)	0.15
		Model 2:	1.00 (Reference)	1.09 (0.68-1.75)	1.60 (0.84-3.04)	0.11	1.00 (Reference)	1.01 (0.70-1.47)	1.37 (0.83-2.28)	0.17
	Index	*Unadjusted:*	1.00 (Reference)	1.18 (0.85-1.64)	1.37 (0.99-1.90)	0.06	1.00 (Reference)	1.13 (0.86-1.48)	1.20 (0.92-1.57)	0.17
		Model 1:	1.00 (Reference)	1.45 (0.99-2.13)	2.23 (1.35-3.68)	0.002	1.00 (Reference)	1.31 (0.97-1.79)	1.64 (1.10-2.45)	0.01
		Model 2:	1.00 (Reference)	1.55 (1.05-2.31)	2.36 (1.38-4.02)	0.002	1.00 (Reference)	1.38 (1.00-1.90)	1.73 (1.13-2.66)	0.01
**Select ratios**										
	Muscle:fascia	*Unadjusted:*	1.00 (Reference)	0.74 (0.54-1.02)	0.69 (0.50-0.95)	0.03	1.00 (Reference)	0.76 (0.59-0.99)	0.67 (0.52-0.87)	0.003
		Model 1:	1.00 (Reference)	0.66 (0.47-0.93)	0.58 (0.40-0.84)	0.005	1.00 (Reference)	0.68 (0.52-0.90)	0.56 (0.41-0.75)	1.7x10-4
		Model 2:	1.00 (Reference)	0.70 (0.49-0.99)	0.66 (0.44-0.98)	0.04	1.00 (Reference)	0.70 (0.53-0.93)	0.57 (0.41-0.80)	9.5x10-4
	Subcutaneous fat:fascia	*Unadjusted:*	1.00 (Reference)	0.65 (0.47-0.90)	0.80 (0.59-1.09)	0.15	1.00 (Reference)	0.67 (0.52-0.88)	0.73 (0.57-0.95)	0.02
		Model 1:	1.00 (Reference)	0.68 (0.47-0.98)	0.84 (0.51-1.38)	0.35	1.00 (Reference)	0.67 (0.49-0.90)	0.72 (0.48-1.06)	0.07
		Model 2:	1.00 (Reference)	0.65 (0.44-0.95)	0.78 (0.46-1.31)	0.24	1.00 (Reference)	0.63 (0.46-0.86)	0.65 (0.43-0.97)	0.02

Hazard ratios (HRs) and 95% confidence intervals (CI) from Cox proportional models. Model 1 adjustments include age, ECOG PS, sex, diabetes, albumin, and BMI in morphomic variable analyses. Model 2 additionally includes CA19-9 and race. BMI Model 1 N: 458 (N progression=212, N death=319); Morphomics Model 1 N: 476 (N progression=222, N death=332). *BMI: Body mass index, ECOG PS: Eastern Cooperative Oncology Group performance status.*

© 2024 Gunchick V et al. JAMA Network Open.

eFigure 2. Association shapes of BMI and morphomic variables with progression-free and overall survival

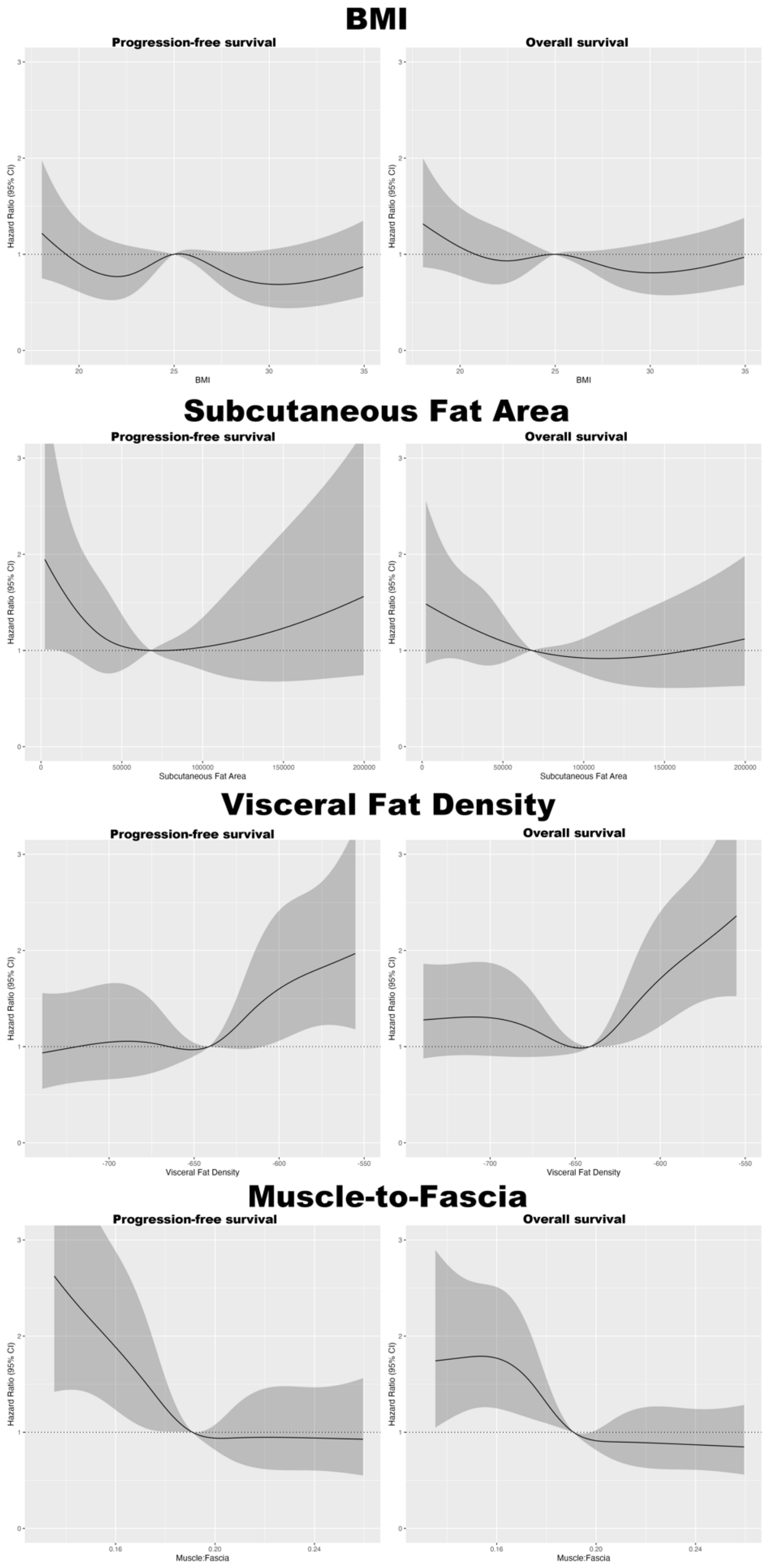

Restricted cubic splines describing relationships between body mass index and select morphomic variables with progression-free and overall survival after adjustment for adjusted for age, ECOG PS, sex, BMI, albumin, and diabetes. *BMI: Body mass index, ECOG PS: Eastern Cooperative Oncology Group performance status.*

© 2024 Gunchick V et al. JAMA Network Open.

eTable 4. Sensitivity analyses

	Progression-free survival HR (95% CI), p-trend				Overall survival HR (95% CI), p-trend			
**BMI sensitivity analysis: all patients with BMI data (BMI≥ 18.5)**								
	**18.5-24.9**	**25.0-29.9**	**≥30**	**p-trend**	**18.5-24.9**	**25.0-29.9**	**≥30**	**p-trend**
	1.00 (Reference)	0.93 (0.69, 1.26)	0.94 (0.65, 1.36)	0.66	1.00 (Reference)	0.88 (0.69, 1.12)	0.90 (0.67, 1.12)	0.37
	**Low, middle, high tertiles, p-trend** HR (95% CI), p-trend							
	1.00 (Reference)	0.97 (0.71, 1.34)	0.80 (0.58, 1.12)	0.20	1.00 (Reference)	0.94 (0.73, 1.23)	0.85 (0.66, 1.10)	0.22
**Morphomic sensitivity analysis 1: T10-T12 vertebral levels**								
Muscle area	1.00 (Reference)	0.54 (0.35-0.85)	0.46 (0.26-0.82)	0.02	1.00 (Reference)	0.63 (0.44-0.92)	0.52 (0.32-0.85)	0.02
Subcutaneous fat area	1.00 (Reference)	0.58 (0.40-0.84)	0.68 (0.41-1.13)	0.07	1.00 (Reference)	0.68 (0.51-0.91)	0.54 (0.36-0.81)	0.003
Visceral fat density	1.00 (Reference)	0.99 (0.71-1.39)	1.53 (1.10-2.13)	0.01	1.00 (Reference)	0.86 (0.65-1.13)	1.33 (1.01-1.75)	0.05
Fascia index	1.00 (Reference)	1.44 (0.99-2.10)	1.85 (1.14-2.98)	0.01	1.00 (Reference)	1.16 (0.86-1.56)	1.23 (0.84-1.79)	0.28
Muscle-to-Fascia	1.00 (Reference)	0.75 (0.54-1.03)	0.68 (0.48-0.97)	0.03	1.00 (Reference)	0.82 (0.63-1.08)	0.69 (0.52-0.92)	0.01
**Morphomic sensitivity analysis 2: exclude BMI from covariate adjustments**								
Muscle area	1.00 (Reference)	0.85 (0.61-1.17)	0.68 (0.48-0.98)	0.04	1.00 (Reference)	0.98 (0.75-1.28)	0.83 (0.62-1.10)	0.20
Subcutaneous fat area	1.00 (Reference)	0.63 (0.45-0.88)	0.86 (0.61-1.21)	0.32	1.00 (Reference)	0.71 (0.54-0.94)	0.78 (0.59-1.03)	0.08
Visceral fat density	1.00 (Reference)	1.12 (0.79-1.58)	1.65 (1.18-2.30)	0.003	1.00 (Reference)	0.92 (0.70-1.21)	1.44 (1.10-1.88)	0.01
Fascia index	1.00 (Reference)	1.22 (0.86-1.71)	1.51 (1.07-2.15)	0.02	1.00 (Reference)	1.17 (0.88-1.54)	1.26 (0.95-1.67)	0.12
Muscle-to-Fascia	1.00 (Reference)	0.68 (0.49-0.94)	0.60 (0.42-0.86)	0.01	1.00 (Reference)	0.70 (0.54-0.92)	0.59 (0.44-0.79)	$4.2x10^{-4}$
**Morphomic sensitivity analysis 3: only venous-phase scans**								
Muscle area	1.00 (Reference)	0.68 (0.46-1.00)	0.67 (0.44-1.02)	0.07	1.00 (Reference)	0.88 (0.64-1.20)	0.83 (0.59-1.17)	0.30
Subcutaneous fat area	1.00 (Reference)	0.59 (0.39-0.88)	0.63 (0.36-1.09)	0.06	1.00 (Reference)	0.70 (0.51-0.96)	0.54 (0.35-0.84)	0.01
Visceral fat density	1.00 (Reference)	0.98 (0.67-1.42)	1.51 (1.03-2.21)	0.03	1.00 (Reference)	0.89 (0.66-1.20)	1.46 (1.07-2.00)	0.02
Fascia index	1.00 (Reference)	1.34 (0.88-2.05)	2.29 (1.33-3.94)	0.003	1.00 (Reference)	1.27 (0.91-1.77)	1.62 (1.06-2.49)	0.03
Muscle-to-Fascia	1.00 (Reference)	0.56 (0.39-0.80)	0.57 (0.38-0.85)	0.01	1.00 (Reference)	0.63 (0.47-0.85)	0.55 (0.40-0.76)	$3.0x10^{-4}$
**Morphomic sensitivity analysis 4: L3 vertebral level**								
Muscle area	1.00 (Reference)	0.91 (0.59-1.40)	0.80 (0.46-1.41)	0.43	1.00 (Reference)	0.98 (0.68-1.40)	0.88 (0.56-1.39)	0.52
Subcutaneous fat area	1.00 (Reference)	0.72 (0.50-1.02)	0.68 (0.42-1.11)	0.10	1.00 (Reference)	0.80 (0.60-1.07)	0.66 (0.45-0.99)	0.04
Subcutaneous fat index	1.00 (Reference)	1.28 (0.84-1.96)	1.34 (0.84-2.14)	0.27	1.00 (Reference)	0.88 (0.63-1.24)	1.04 (0.72-1.51)	0.63
Visceral fat density	1.00 (Reference)	1.02 (0.72-1.45)	1.53 (1.09-2.15)	0.01	1.00 (Reference)	0.92 (0.70-1.21)	1.46 (1.11-1.93)	0.01
Fascia index	1.00 (Reference)	1.47 (1.02-2.11)	1.92 (1.21-3.05)	0.005	1.00 (Reference)	1.31 (0.97-1.76)	1.59 (1.09-2.30)	0.01
Muscle-to-Fascia	1.00 (Reference)	0.75 (0.53-1.06)	0.66 (0.45-0.97)	0.04	1.00 (Reference)	0.69 (0.52-0.91)	0.56 (0.41-0.77)	$3.7x10^{-4}$

Hazard ratios (HRs) and 95% confidence intervals (CI) from multivariate Cox proportional models. BMI sensitivity analysis and morphomic sensitivity analysis 2 models were adjusted for age, ECOG PS, sex, diabetes, and blood albumin. Sensitivity analysis 1 and 3 additionally adjusted for BMI. *BMI: Body mass index, ECOG PS: Eastern Cooperative Oncology Group performance status.*

© 2024 Gunchick V et al. JAMA Network Open.

eTable 5. Comparing patient characteristics between the highest and lowest morphomic variable tertiles

Variable	Lowest tertile, highest tertile<br>Median [25%, 75%] or N%, p-value								
	Subcutaneous Fat Area			Visceral Fat Density			Muscle-to-Fascia		
**Demographics**									
Age	63 [57, 67]	61 [55, 67]	0.19	61 [54, 66]	63 [58, 68]	0.004	66 [60, 70]	59 [51, 64]	<0.001
Sex (Male)	119 (74.8)	71 (44.9)	<0.001	98 (62.4)	86 (5.5)	0.23	75 (47.2)	107 (67.7)	<0.001
Baseline CA19-9	1478 [172, 10000]	1902 [176, 11540]	0.92	1140 [87, 9948]	2357 [227, 15539]	0.08	2258 [218, 11793]	733 [120, 6383]	0.05
ECOG PS (1)	94 (59.1)	83 (52.5)	0.29	81 (51.6)	78 (4.9)	0.87	87 (54.7)	78 (49.4)	0.40
**Baseline comorbidities**									
Diabetes	37 (23.3)	54 (34.2)	0.04	44 (28)	42 (2.7)	0.93	57 (35.8)	33 (20.9)	0.005
PDA Resection	2 (1.3)	2 (1.3)	1.00	1 (0.6)	6 (0.4)	0.12	3 (1.9)	1 (0.6)	0.62
Hypertension	61 (38.4)	94 (59.5)	<0.001	77 (49)	72 (4.6)	0.69	98 (61.6)	57 (36.1)	<0.001
Abdominal pain	95 (59.7)	100 (63.3)	0.59	99 (63.1)	92 (5.8)	0.53	96 (60.4)	95 (60.1)	1.00
Hyperlipidemia	20 (12.6)	33 (20.9)	0.07	33 (21)	27 (1.7)	0.49	38 (23.9)	25 (15.8)	0.10
Osteoarthritis	3 (1.9)	15 (9.5)	0.007	12 (7.6)	5 (0.3)	0.14	11 (6.9)	1 (0.6)	0.008
Gastro reflux	29 (18.2)	63 (39.9)	<0.001	47 (29.9)	44 (2.8)	0.83	57 (35.8)	26 (16.5)	<0.001
Albumin (N-H)	136 (85.5)	132 (83.5)	0.74	139 (88.5)	125 (7.9)	0.06	129 (81.1)	138 (87.3)	0.17
**Anthropometrics**									
BMI	21.9 [19.9, 23.4]	29.7 [27.3, 33.2]	<0.001	27.1 [23.8, 32.0]	23.0 [21.1, 25.9]	<0.001	27.0 [22.8, 30.3]	23.8 [21.8, 26.5]	<0.001
Height (cm)	171 [165, 178]	170 [160, 178]	0.10	172 [164, 179]	168 [160, 176]	0.006	168 [160, 176]	171 [164, 178]	0.049
Weight (kg)	64.0 [55.7, 71.8]	85.1 [74.9, 101.9]	<0.001	82 [67, 95]	65 [57, 75]	<0.001	77 [64, 90]	69 [61, 80]	0.001
BSA	1.74 [1.60, 1.89]	1.99 [1.83, 2.20]	<0.001	1.98 [1.79, 2.18]	1.76 [1.60, 1.90]	<0.001	1.89 [1.70, 2.09]	1.81 [1.66, 1.97]	0.008

Chi-square or Fisher's exact test was used to compare categorical variables and ANOVA or the Kruskal-Wallis test for continuous variables, as appropriate. *BMI: body mass index, BSA: body surface area, CA19-9: cancer antigen 19-9, ECOG PS: Eastern Cooperative Oncology Group performance status, Gastro: gastroesophageal, HU: Hounsfield units, PDA: pancreatic ductal adenocarcinoma.*

© 2024 Gunchick V et al. JAMA Network Open.

eFigure 3. Association of key morphomic variables' area and density variables

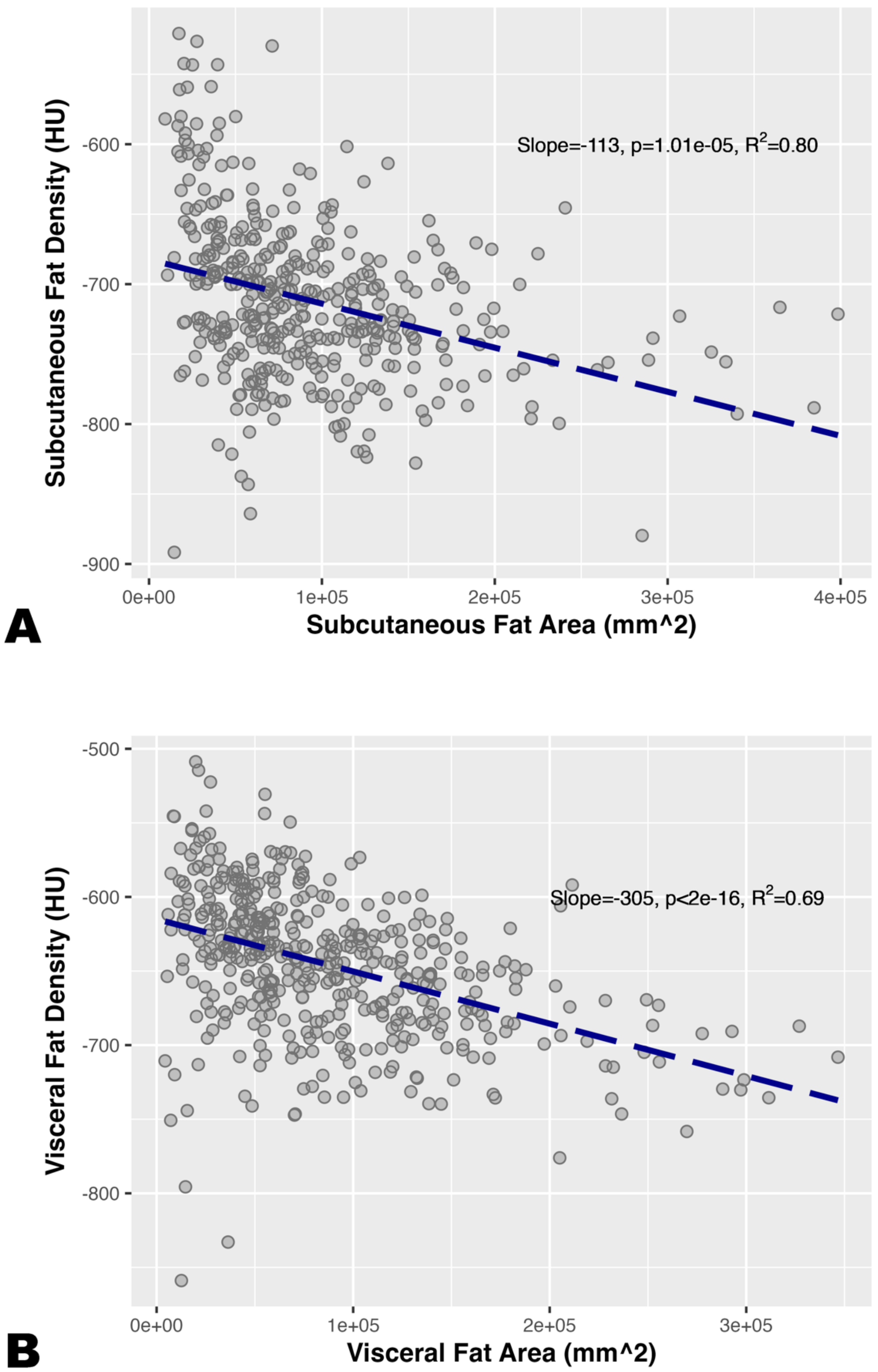

A scatter plot displaying key morphomic variables' area versus density variables with results (slope, p-value, and R^2) from linear regression of area regressed on density adjusted for age, ECOG PS, sex, BMI, albumin, and diabetes. *BMI: Body mass index, ECOG PS: Eastern Cooperative Oncology Group performance status.*

© 2024 Gunchick V et al. JAMA Network Open.

eFigure 4. Associations of metabolites and morphomic variables

Metabolites grouped by morphomic variable	Chemical Formula	HMDB	Median Concentration [25%, 75%]	β (95% CI)	p-value
**Muscle-to-Fascia**					
3-Hydroxybutyrylcarnitine	C11H21NO5	HMDB13127	2918919.4 [1762748.1, 4912718.3]	-0.20 (-0.31 - -0.08)	8.2x10-4
C4H6O3(3)	C4H6O3	N/A	99534264.3 [83788968, 120513322.8]	-0.17 (-0.29 - -0.05)	0.005
(R)-2-hydroxy-4-methylpentanoate	C6H12O3	HMDB00624	16887958.8 [13193883.8, 20939539.3]	-0.17 (-0.29 - -0.05)	0.005
D-Glucosamine	C6H13NO5	HMDB01514	29514713.4 [23165586.3, 40703514.2]	-0.17 (-0.28 - -0.05)	0.006
3-methyl-2-oxopentanoate/4-methyl-2-oxopentanoate(ketoleucine)	C6H10O3	HMDB00491	475061003.4 [394103896.9, 554728638.2]	-0.17 (-0.28 - -0.05)	0.006
L-erythrulose	C4H8O4	HMDB06293	77048108.7 [62823077.8, 95076416.1]	-0.17 (-0.28 - -0.05)	0.007
2-methylglutaconic acid	C6H8O4	HMDB02266	91429380.2 [74480085, 112528058.7]	-0.16 (-0.28 - -0.04)	0.008
octanoyl carnitine/L-Octanoylcarnitine	C15H29NO4	HMDB00791	6473204.8 [4379431.5, 9577853.8]	-0.16 (-0.27 - -0.05)	0.006
decanoyl carnitine	C17H33NO4	HMDB06210	8481124.9 [5597382.4, 12975824.6]	-0.16 (-0.27 - -0.04)	0.007
alpha-D-glucose	C6H12O6	HMDB03345	706782244.5 [580520917.4, 871579995.9]	-0.15 (-0.27 - -0.03)	0.01
decenoylcarnitine	C17H31NO4	HMDB13205	6743941.1 [5100748.8, 9554051]	-0.15 (-0.26 - -0.03)	0.01
O-acetylcarnitine	C9H17NO4	HMDB00201	549256248.8 [422608556.4, 754124813.3]	-0.14 (-0.25 - -0.03)	0.01
Elaidic carnitine/Vaccenyl carnitine	C25H47NO4	HMDB06464	1707781.4 [1346351, 2207080.9]	-0.14 (-0.25 - -0.02)	0.02
tetradecanoyl carnitine(myristoyl carnitine)	C21H41NO4	HMDB05066	432119.8 [309377.9, 578516.7]	-0.13 (-0.25 - -0.02)	0.02
Cystine	C6H12N2O4S2	HMDB00192	46074584.2 [37527291.4, 58039934.6]	-0.12 (-0.24 - -0.01)	0.04
4(R)-hydroxy-dodec-6Z-enoate/4(S)-hydroxy-dodec-6Z-enoate	C12H22O3	N/A	2291708.3 [1658582.9, 2969365.7]	-0.12 (-0.23 - 0.00)	0.04
lauroyl carnitine	C19H37NO4	HMDB02250	1899775.1 [1281228.2, 2700104.9]	-0.11 (-0.23 - 0.00)	0.05
creatinine	C4H7N3O	HMDB00562	3166524198 [2760763076.5, 3715156362.5]	0.12 (0.00 - 0.23)	0.04
Glycerol 3-phosphate	C3H9O6P	HMDB00126	765434 [487128.8, 1091510.9]	0.12 (0.00 - 0.23)	0.04
C27H44O2(5)	C27H44O2	N/A	183046.1 [136790.7, 267916.7]	0.12 (0.01 - 0.23)	0.04
L-arabinitol/xylitol/D-ribitol	C5H12O5	N/A	10831754.7 [9450588.7, 13424725.5]	0.12 (0.01 - 0.24)	0.04
Nicotinamide	C6H6N2O	HMDB01406	938688.3 [709710.4, 1321063.6]	0.12 (0.01 - 0.24)	0.03
acetylcholine/4-(trimethylammonio)butanoate	C7H15NO2	HMDB00895	72212500.3 [59174559, 87979003.9]	0.12 (0.00 - 0.25)	0.05
C27H42O4(3)	C27H42O4	N/A	418604 [339791.8, 536248.4]	0.13 (0.01 - 0.24)	0.03
Methylisocitric acid/2-methylcitrate	C7H10O7	HMDB06471	1964513.2 [1602682.3, 2323291.3]	0.13 (0.01 - 0.24)	0.03
kynurenate	C10H7NO3	HMDB00715	783460.1 [547204.4, 1071163.9]	0.13 (0.01 - 0.24)	0.03
Methylimidazoleacetic acid	C6H8N2O2	HMDB02820	8897461.3 [5137510.8, 15253640.4]	0.13 (0.01 - 0.24)	0.03
Glycylproline/L-Prolinylglycine	C7H12N2O3	HMDB00721	966664.8 [769766.6, 1283390.7]	0.13 (0.02 - 0.25)	0.02
itaconate/Mesaconate	C5H6O4	HMDB02092	11324216.7 [9801489.8, 13186140.4]	0.14 (0.02 - 0.25)	0.02
L-tryptophan	C11H12N2O2	HMDB00929	368276238.8 [300710461.7, 427049555.3]	0.14 (0.01 - 0.27)	0.04
3-Hydroxy-N6N6N6-trimethyl-L-lysine	C9H20N2O3	HMDB01422	1736319.4 [1442653, 2214621]	0.14 (0.03 - 0.26)	0.02
glycine	C2H5NO2	HMDB00123	26384524.1 [22377229.2, 31640614.7]	0.14 (0.02 - 0.26)	0.02
spermidine dialdehyde-2	C7H13NO2	HMDB13076	140833131.1 [40033912.7, 595146810]	0.14 (0.03 - 0.26)	0.01
choline phosphate	C5H14NO4P	HMDB01565	385008.2 [190308.5, 813818.4]	0.15 (0.03 - 0.26)	0.01
guanosine	C10H13N5O5	HMDB00133	8296001.7 [6300087.2, 10539740.7]	0.15 (0.02 - 0.27)	0.02
lysine	C6H14N2O2	HMDB00182	157589882.9 [130731825.1, 182919500.3]	0.15 (0.03 - 0.27)	0.01
L-threonate	C4H8O5	HMDB00943	136918725.3 [115260338.8, 159151148.2]	0.15 (0.04 - 0.27)	0.008
[13C]3lactate	3[13C]C3H6O3	N/A	29625591746.5 [28658891742.8, 31288441989.2]	0.15 (0.04 - 0.27)	0.008
N-acetyl-L-aspartate/2-Amino-3-oxoadipate	C6H9NO5	HMDB00812	5679253 [4933608.5, 6575864]	0.16 (0.04 - 0.27)	0.007
L-proline	C5H9NO2	HMDB00162	1186662515 [963032863.6, 1489398603.2]	0.16 (0.05 - 0.27)	0.005
5,6-dihydrothymine	C5H8N2O2	HMDB00079	169450810.6 [122051090.3, 235985341.4]	0.16 (0.04 - 0.28)	0.008
Riboflavin	C17H20N4O6	HMDB00244	243597 [162347.4, 378893.5]	0.17 (0.05 - 0.28)	0.005
inosine	C10H12N4O5	HMDB00195	7810561.6 [4003945.1, 18053158.1]	0.17 (0.05 - 0.28)	0.004
L-fucose/L-Fuculose	C6H12O5	HMDB00174	2307453.9 [1342305.8, 3583668.1]	0.17 (0.05 - 0.28)	0.004
2-deoxy-D-ribose	C5H10O4	HMDB03224	290872 [232344, 344939]	0.17 (0.05 - 0.28)	0.005
L-xylonate/L-lyxonate	C5H10O6	HMDB60256	20573382.9 [16917459.8, 26560126.2]	0.17 (0.05 - 0.28)	0.004
sarcosine	C3H7NO2	HMDB00271	614169 [502969.5, 805573.8]	0.18 (0.07 - 0.30)	0.001
glutathione	C10H17N3O6S	HMDB00125	19239.1 [9663.5, 35996]	0.20 (0.07 - 0.32)	0.003
sn-Glycero-3-phosphocholine	C8H20NO6P	HMDB00086	111472511.8 [87050802.7, 147206133.8]	0.22 (0.10 - 0.33)	*3.3x10-4
citr_L[e]	C6H13N3O3	N/A	90464834.9 [69619277.2, 116760379]	0.22 (0.11 - 0.34)	*1.1x10-4
[13C]6alpha-D-glucose	6[13C]C6H12O6	N/A	444918658.9 [421782882.6, 474052356.2]	0.24 (0.13 - 0.35)	*3.2x10-5
acetylcarnosine	C11H16N4O4	HMDB12881	1257321.8 [839372.5, 1746610.4]	0.34 (0.21 - 0.47)	*1.3x10-6
**Subcutaneous Fat Area**					
2-keto-4-methylthiobutyrate	C5H8O3S	HMDB01553	58320 [27203.9, 105308.9]	-0.10 (-0.18 - -0.03)	0.006
5-Methoxyindoleacetate	C11H11NO3	HMDB04096	3629530.1 [2779856.2, 4686816.3]	-0.10 (-0.17 - -0.03)	0.003
L-fucose/L-Fuculose	C6H12O5	HMDB00174	2307453.9 [1342305.8, 3583668.1]	-0.08 (-0.15 - -0.02)	0.007
7,9-dihydro-1H-purine-2688(3H)-trione	C5H4N4O3	N/A	668551246 [541891638.6, 812140478.4]	-0.08 (-0.15 - -0.02)	0.02
citr_L[e]	C6H13N3O3	N/A	90464834.9 [69619277.2, 116760379]	-0.08 (-0.14 - -0.02)	0.01
acetylcarnosine	C11H16N4O4	HMDB12881	1257321.8 [839372.5, 1746610.4]	-0.08 (-0.16 - 0.00)	0.05
N6N6N6-Trimethyl-L-lysine	C9H20N2O2	HMDB01325	20792565.4 [16722368.2, 27227296.6]	-0.08 (-0.14 - -0.01)	0.03
L-xylonate/L-lyxonate	C5H10O6	HMDB60256	20573382.9 [16917459.8, 26560126.2]	-0.08 (-0.14 - -0.01)	0.02
L-Iditol/D-glucitol/galactitol	C6H14O6	N/A	11280189.4 [4977002.9, 36269199.4]	-0.08 (-0.14 - -0.01)	0.02
L-arabinitol/xylitol/D-ribitol	C5H12O5	N/A	10831754.7 [9450588.7, 13424725.5]	-0.07 (-0.14 - -0.01)	0.03
Trimethylamine N-oxide	C3H9NO	HMDB00925	69251573.8 [39396776.7, 135193609.4]	-0.07 (-0.13 - -0.01)	0.02
quinolinate	C7H5NO4	HMDB00232	1296084.1 [1001356.8, 1695587.5]	-0.07 (-0.14 - 0.00)	0.04
L-threonate	C4H8O5	HMDB00943	136918725.3 [115260338.8, 159151148.2]	-0.07 (-0.13 - 0.00)	0.04
4-Hydroxyphenylacetate/2-Hydroxyphenylacetate/3,4-Dihydroxyphenylacetaldehyde	C8H8O3	HMDB00020	3091846.7 [2257387.4, 4828084.9]	-0.07 (-0.13 - 0.00)	0.05
ribose-phosphate	C5H11O8P	HMDB01548	175802 [108707.7, 277116.7]	-0.06 (-0.13 - 0.00)	0.04
clupanodonic acid/docosa-4,7,10,13,16-pentaenoic acid	C22H34O2	HMDB06528	9166704.4 [5659782.2, 14617121.5]	0.06 (0.00 - 0.13)	0.05
Hypotaurine	C2H7NO2S	HMDB00965	526884.6 [355046.6, 706189.1]	0.07 (0.00 - 0.13)	0.04
docosahexaenoate(DHA)	C22H32O2	HMDB02183	17494128.7 [11156280, 23833930.4]	0.07 (0.00 - 0.13)	0.04
Eicosenoic acid	C20H38O2	HMDB02231	12752394.2 [8180874.9, 18482985.9]	0.07 (0.01 - 0.13)	0.03
oleic acid/elaidate/trans-vaccenate	C18H34O2	HMDB00207	1646764141 [1120202986.8, 2453283528.2]	0.07 (0.01 - 0.14)	0.03
C4H9NO2(4)	C4H9NO2	N/A	45346509.1 [36891092.5, 54331276.7]	0.07 (0.01 - 0.13)	0.02
palmitoleate	C16H30O2	HMDB03229	170098847.1 [99484814.7, 303447712.9]	0.07 (0.01 - 0.14)	0.03
6(S)-hydroxy-tetradeca-2E4E8Z-trienoate/6(R)-hydroxy-tetradeca-2E4E8Z-trienoate	C14H22O3	N/A	1523148.2 [1042777.8, 1944106.6]	0.07 (0.01 - 0.14)	0.02
palmitate	C16H32O2	HMDB00220	1011105689.5 [665584462.9, 1454752036.8]	0.07 (0.01 - 0.14)	0.02
pentadecanoate	C15H30O2	HMDB00826	16536936.2 [10263818.3, 22438829.7]	0.08 (0.01 - 0.14)	0.02
O-acetylcarnitine	C9H17NO4	HMDB00201	549256248.8 [422608556.4, 754124813.3]	0.08 (0.01 - 0.14)	0.02
tetradecanoyl carnitine(myristoyl carnitine)	C21H41NO4	HMDB05066	432119.8 [309377.9, 578516.7]	0.08 (0.02 - 0.14)	0.01
arachidonic acid	C20H32O2	HMDB01043	53837322.7 [38727562.4, 71576052]	0.08 (0.02 - 0.15)	0.01
L-palmitoylcarnitine	C23H45NO4	HMDB00222	1134272.7 [825489.8, 1443339.4]	0.08 (0.02 - 0.14)	0.007
C14H24O3(4)	C14H24O3	N/A	1258540 [959358.9, 1678052.2]	0.09 (0.02 - 0.15)	0.008
trans-Hexadec-2-enoyl carnitine	C23H43NO4	HMDB06317	395272.9 [248669.4, 602682.9]	0.09 (0.02 - 0.15)	0.008
stearoylcarnitine	C25H49NO4	HMDB00848	264873.3 [202914.7, 343003.3]	0.09 (0.02 - 0.15)	0.007
4(R)-hydroxy-dodec-6Z-enoate/4(S)-hydroxy-dodec-6Z-enoate	C12H22O3	N/A	2291708.3 [1658582.9, 2969365.7]	0.09 (0.03 - 0.15)	0.005
stearate	C18H36O2	HMDB00827	260562335.5 [201164509.5, 336025095.6]	0.10 (0.03 - 0.16)	0.003
3-Hydroxybutyrylcarnitine	C11H21NO5	HMDB13127	2918919.4 [1762748.1, 4912718.3]	0.10 (0.03 - 0.16)	0.003
Linoleyl carnitine/Linoelaidyl carnitine	C25H45NO4	HMDB06469	1029316.8 [799448.3, 1333747.5]	0.10 (0.04 - 0.16)	0.002
lauroyl carnitine	C19H37NO4	HMDB02250	1899775.1 [1281228.2, 2700104.9]	0.10 (0.04 - 0.16)	0.002
Elaidic carnitine/Vaccenyl carnitine	C25H47NO4	HMDB06464	1707781.4 [1346351, 2207080.9]	0.10 (0.04 - 0.16)	0.001
margarate	C17H34O2	HMDB02259	19635924.9 [13848683.8, 27511183.4]	0.10 (0.04 - 0.16)	0.001
decenoylcarnitine	C17H31NO4	HMDB13205	6743941.1 [5100748.8, 9554051]	0.12 (0.05 - 0.18)	*2.7x10-4
decanoylcarnitine	C17H33NO4	HMDB06210	8481124.9 [5597382.4, 12975824.6]	0.12 (0.06 - 0.18)	*1.2x10-4
octanoylcarnitine/L-Octanoylcarnitine	C15H29NO4	HMDB00791	6473204.8 [4379431.5, 9577853.8]	0.13 (0.07 - 0.19)	*3.4x10-5
pristanic acid	C19H38O2	HMDB00795	775307.1 [562180.2, 1047941.5]	0.15 (0.08 - 0.21)	*5.7x10-6
**Visceral Fat Density**					
acetylcarnosine	C11H16N4O4	HMDB12881	1257321.8 [839372.5, 1746610.4]	-0.17 (-0.32 - -0.02)	0.03
3-methyl-2-oxopentanoate/4-methyl-2-oxopentanoate(ketoleucine)	C6H10O3	HMDB00491	475061003.4 [394103896.9, 554728638.2]	-0.16 (-0.29 - -0.03)	0.01
Valine	C5H11NO2	HMDB00883	396712209.2 [322740827.8, 447735353.6]	-0.16 (-0.29 - -0.03)	0.01
Xanthine	C5H4N4O2	HMDB00292	60549627.5 [46855699.2, 74201351.7]	-0.13 (-0.26 - -0.01)	0.03
Glycyl-phenylalanine	C11H14N2O3	HMDB28848	614730.2 [331127, 1001484.8]	-0.13 (-0.25 - 0.00)	0.04
8,11,14-Eicosatrienoic acid (DGLA)	C20H34O2	HMDB02925	12821000.4 [8822839.7, 17650074.2]	0.13 (0.00 - 0.25)	0.05
3-Deoxy-D-glycero-D-galacto-2-nonulosonic acid	C9H16O9	HMDB00425	1287688.9 [1124110.9, 1513525.1]	0.13 (0.01 - 0.26)	0.04
quinolinate	C7H5NO4	HMDB00232	1296084.1 [1001356.8, 1695587.5]	0.14 (0.01 - 0.27)	0.04
myo-inositol	C6H12O6	HMDB00211	50885904.6 [43718873.7, 59901604]	0.15 (0.02 - 0.28)	0.02
L-arabinitol/xylitol/D-ribitol	C5H12O5	N/A	10831754.7 [9450588.7, 13424725.5]	0.15 (0.03 - 0.28)	0.02
L-Alanyl-L-leucine	C9H18N2O3	HMDB28691	6528196.2 [5059259.8, 9168037.5]	0.20 (0.07 - 0.32)	0.002

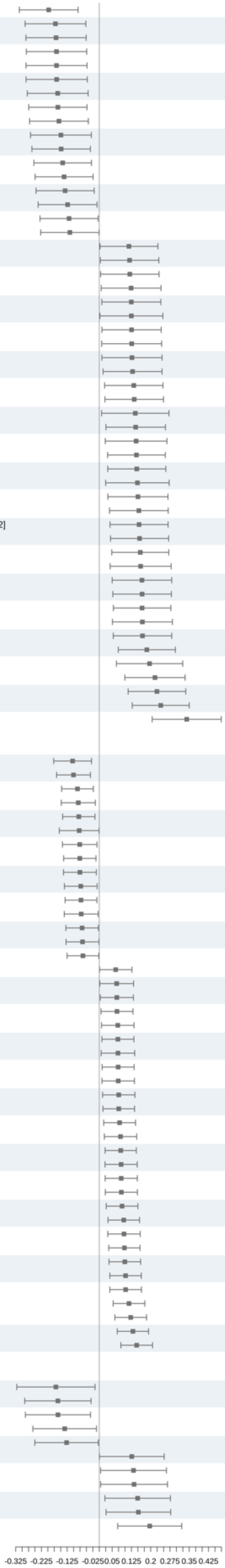

Multivariate linear regression of metabolites (log-transformed (ln[x+1]) and z-scored) and morphomic variables (z-scored). Models were adjusted for age, ECOG PS, sex, BMI, albumin, and diabetes. All associations with p<0.05 are shown. *BMI: Body mass index, CI: confidence interval, ECOG PS: Eastern Cooperative Oncology Group performance status. *Indicates significance per our p<0.00063 cutoff.*

© 2024 Gunchick V et al. JAMA Network Open.
