# Supplementary material for: Morphomics, Survival, and Metabolites in Patients With Metastatic Pancreatic Cancer
Source: JAMA Netw Open. 2024 Oct 17;7(10):e2440047. doi: 10.1001/jamanetworkopen.2024.40047 (PMC11581562; doi:10.1001/jamanetworkopen.2024.40047)
Supplement: Supplement 2. — Data Sharing Statement [file jamanetwopen-e2440047-s002.pdf]

Data Sharing Statement

Gunchick. Morphomics Associations With Survival and Metabolites in Patients With Metastatic Pancreatic Cancer. *JAMA Netw Open*. Published October 17, 2024.
doi:10.1001/jamanetworkopen.2024.40047

Data
Data available: Yes
Data types: Other (please specify)
Additional Information: Patient, scans and metabolite data Analytic Morphomic body composition measurements
How to access data: More information on Analytic Morphomic body composition measurements used for this study can be found in the online data dictionary (http://www.med.umich.edu/surgery/morphomics/data_dictionary). Cornerstone Pharmaceuticals may be contacted at Vikram.Katragadda@cornerstonpharma.com with interest in trial, scans and metabolite data.
When available: With publication

Supporting Documents
Document types: None

Additional Information
Who can access the data: Researchers whose proposed use of the data has been approved by Cornerstone Pharmaceuticals.
Types of analyses: As per agreement with Cornerstone Pharmaceuticals
Mechanisms of data availability: As per agreement with Cornerstone Pharmaceuticals
